# Using Mathematical Modelling to Explore Hypotheses about the Role of Bovine Epithelium Structure in Foot-And-Mouth Disease Virus-Induced Cell Lysis

**DOI:** 10.1371/journal.pone.0138571

**Published:** 2015-10-02

**Authors:** Kyriaki Giorgakoudi, Simon Gubbins, John Ward, Nicholas Juleff, Zhidong Zhang, David Schley

**Affiliations:** 1 The Pirbright Institute, Pirbright, Surrey, United Kingdom; 2 Department of Mathematical Sciences, Loughborough University, Loughborough, Leicestershire, United Kingdom; 3 State Key Laboratory of Veterinary Etiological Biology, Lanzhou Veterinary Research Institute, Chinese Academy of Agricultural Sciences, Lanzhou, Gansu, China; University Medicine Greifswald, GERMANY

## Abstract

Foot-and-mouth disease (FMD) is a highly contagious disease of cloven-hoofed animals. FMD virus (FMDV) shows a strong tropism for epithelial cells, and FMD is characterised by cell lysis and the development of vesicular lesions in certain epithelial tissues (for example, the tongue). By contrast, other epithelial tissues do not develop lesions, despite being sites of viral replication (for example, the dorsal soft palate). The reasons for this difference are poorly understood, but hypotheses are difficult to test experimentally. In order to identify the factors which drive cell lysis, and consequently determine the development of lesions, we developed a partial differential equation model of FMDV infection in bovine epithelial tissues and used it to explore a range of hypotheses about epithelium structure which could be driving differences in lytic behaviour observed in different tissues. Our results demonstrate that, based on current parameter estimates, epithelial tissue thickness and cell layer structure are unlikely to be determinants of FMDV-induced cell lysis. However, differences in receptor distribution or viral replication amongst cell layers could influence the development of lesions, but only if viral replication rates are much lower than current estimates.

## Introduction

Foot-and-mouth disease (FMD) is one of the most infectious diseases of cloven-hoofed animals [1]. Domestic and wildlife species are susceptible to infection by FMD virus (FMDV), including cattle, swine, sheep, deer, bison and antelope [2]. FMD is of significant worldwide socio-economic importance [1, 3, 4], because it can cause substantially reduced productivity in domestic animals for an extended period of time [1] and has been associated with abortion in pregnant animals and myocarditis and death in young livestock [5].

**Fig 1:**
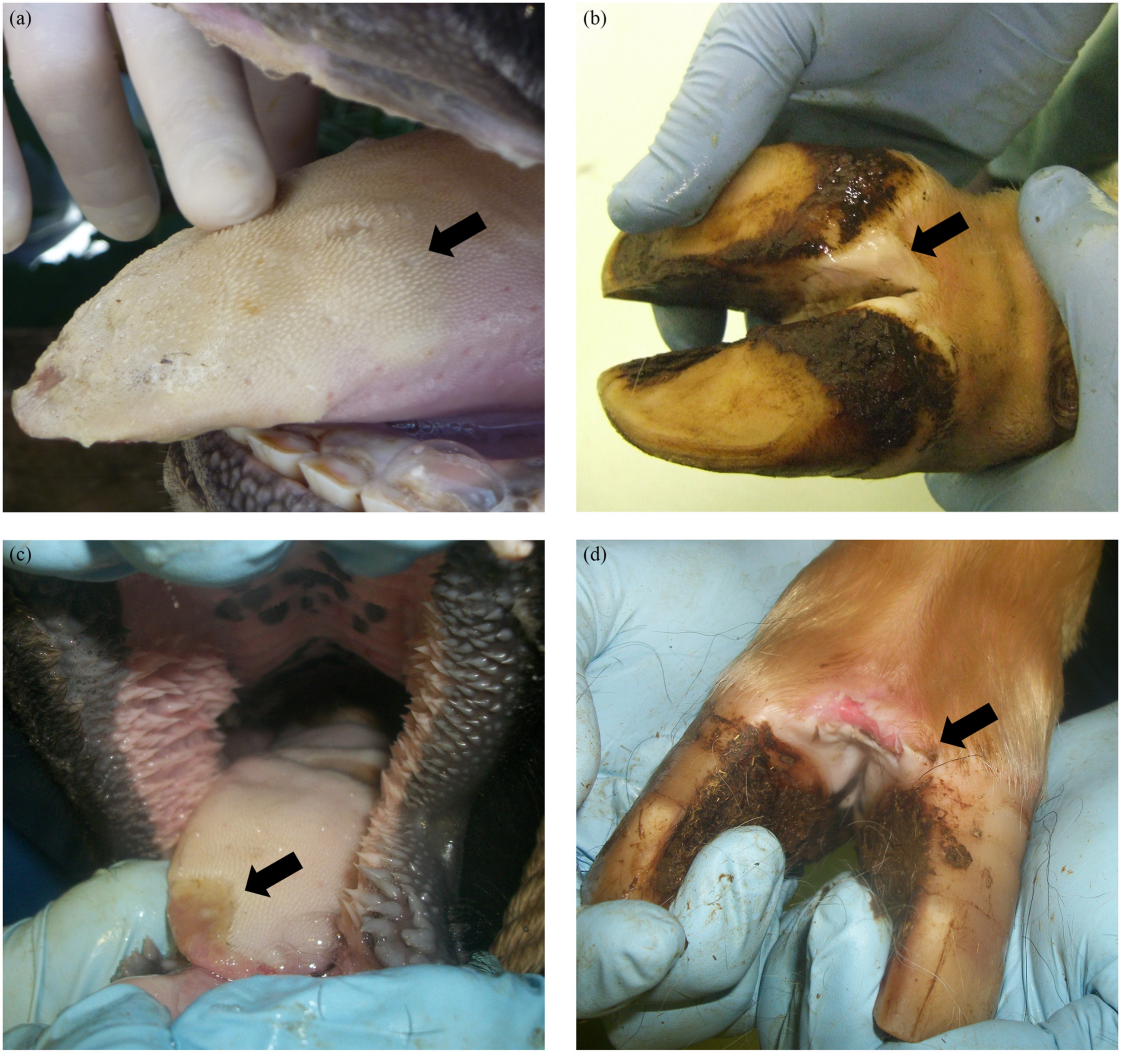
(a)–(d) Typical FMDV epithelial vesicles on the tongue and hoof of infected cattle (black arrows). (a) Recently ruptured tongue vesicle, lesion is extensive as demarcated by blanching of the epithelium across the rostral surface of the tongue. Blanched epithelium has begun to slough, leaving erosions. (b) Unruptured fluid filled vesicle on the heel of the hoof. (c) Erosion on the rostal tip of the tongue, epithelium has sloughed exposing raw epithelium below. (d) Ruptured interdigital vesicle, blanched epithelium is sloughing exposing raw epithelium below.

**Fig 2:**
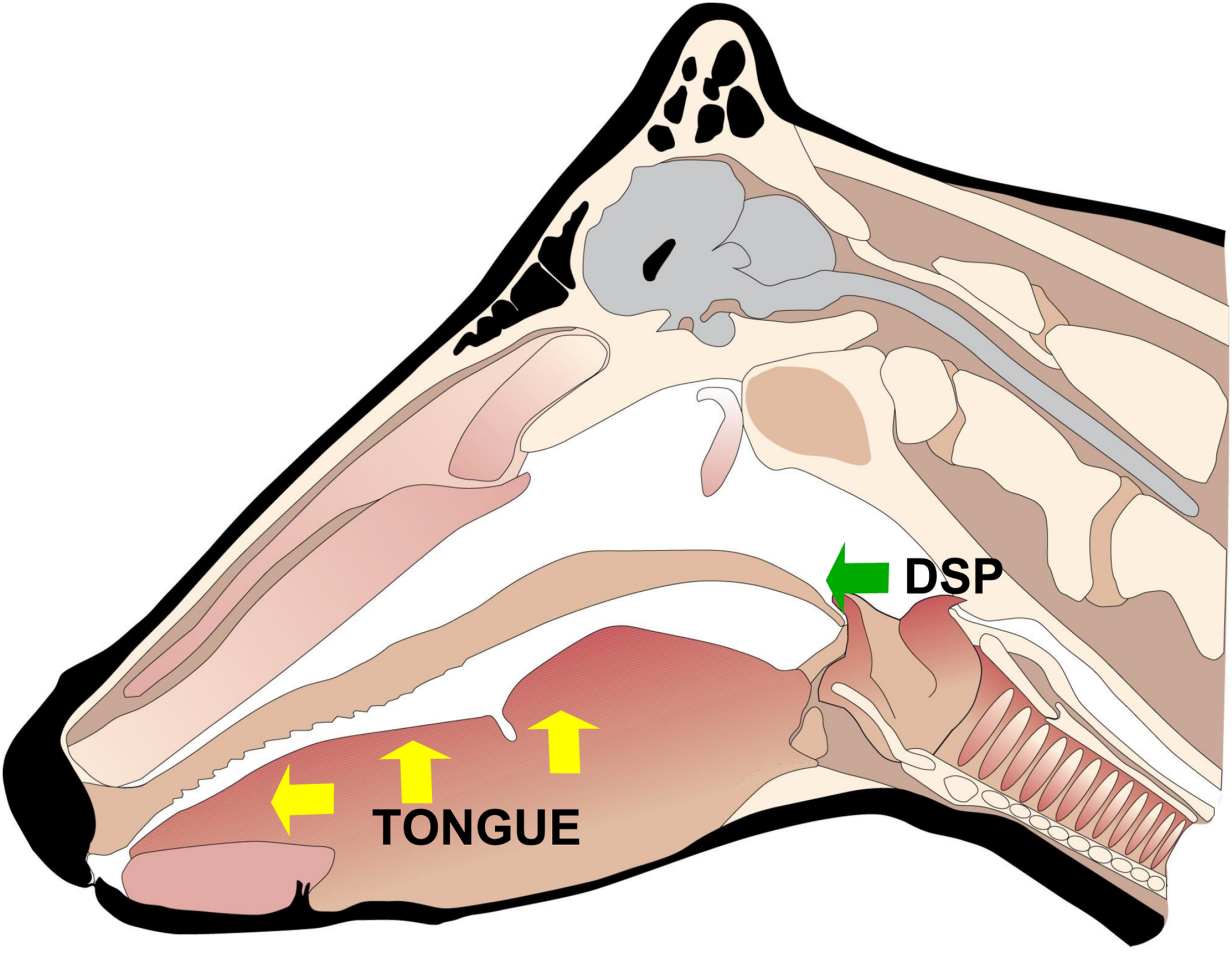
Diagram of cattle head. Location of DSP (green arrow) and tongue (yellow arrows) bovine tissues. Lesions in tongue usually occur close to the tip (left-most yellow arrow).

The principal clinical signs of FMD are vesicular lesions on the feet and in or around the mouth (Fig 1); other clinical signs include oral or nasal discharge, lameness, reluctance to stand or move and fever [5]. The development of vesicular lesions is observed in certain epithelial tissues within infected animals, while other tissues remain unaffected. For example, although cattle develop severe vesicular lesions in the tongue [1], the epithelial layer on the dorsal surface of the soft palate (DSP) (see Fig 2) does not develop visible vesicles or lesions [5]; it is, however, not known whether cell death still occurs within the DSP. The absence of lesions in the DSP is despite the fact that this is considered to be a primary site of infection and one of the main sites of initial FMDV replication [5, 6]. The causes of the different pathological behaviour between the tongue and the DSP are currently unknown, but it has been suggested that it is a consequence of the different epithelial structure of these tissues [5].

Epithelia in both the tongue and DSP are stratified into layers (called basal, spinous, granular and corneal [7]) (see Fig 2(a) in [8]), but the structure of the tissues differs greatly. While the tongue is thick, mainly due to a vast spinous layer, the DSP is much thinner. In addition, the tongue includes all four cell layers, while the DSP lacks distinct granular and corneal layers. Expression levels of the main receptor used by FMDV for cell entry, αvβ6, differ markedly between tongue and DSP, with high levels of expression in tongue and no detectable expression in DSP [9]. There are also differences in expression of αvβ6 between layers within tissues, with the highest levels seen in the spinous layer [9]. Alternatively, viral replication rates could differ between the tissues or between layers in the same tissue. Any or all of these differences could potentially explain the difference in outcome following FMDV infection of the tongue and DSP.

To test experimentally whether or not these differences (in structure, receptor distribution or viral replication) explain why lesions form in the tongue but not in the DSP would be extremely difficult. Accordingly, we developed a partial differential equation (PDE) model to describe dynamics of FMDV in structured epithelium. The model is designed so that it is capable of incorporating the hypothesised differences between tongue and DSP and, hence, can be used to determinine which are consistent with the observed behaviour (i.e. lesions forming in tongue, but not in DSP). Here we focus on establishing why a qualitative difference in the extent of cell death between DSP and tongue exists, and we have thus not embarked on a quantitative estimation of the depth of lesions. The model was parameterised using data from the published literature, with data gaps on epithelium structure filled by new experimental data. The sensitivity of the model predictions to changes in the parameters was explored to assess the robustness of any conclusions.

## Methods

### Mathematical model

**Fig 3:**
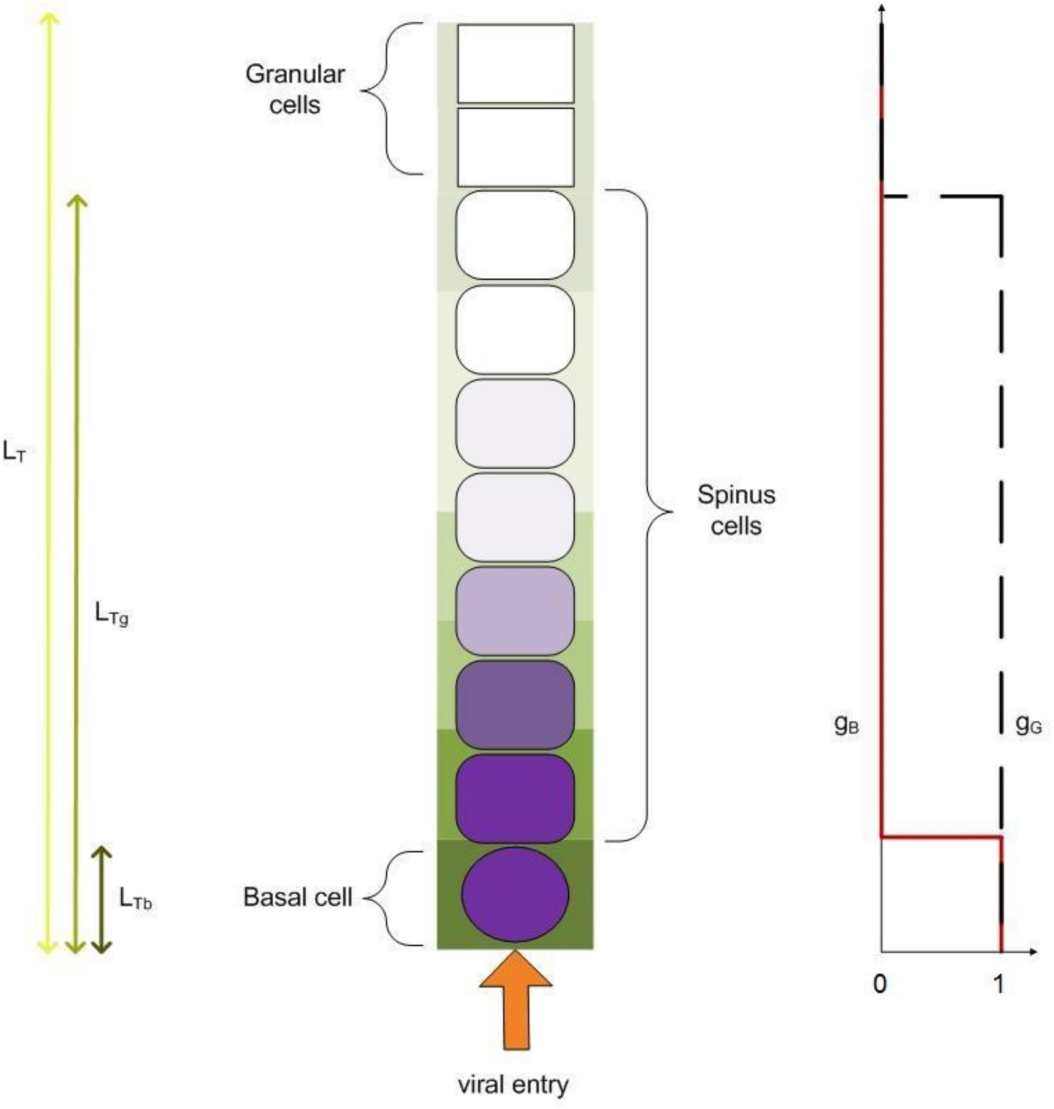
Schematic diagram of the continuum model. Example of FMDV infection of tongue epithelial cells with virus entering through the basement membrane. Darker shades of green and purple indicate higher concentration of extracellular and cellular FMDV respectively. Infection dynamics are the same in DSP, though tissue structure and usual site of viral entry are different. Tongue epithelium thickness is *L*_*T*_, basal-spinous epithelium thickness is *L*_*T*_*g*__, while basal cell layer thickness is *L*_*T*_*b*__ (left hand side). For DSP the equivalents are *L*_*P*_ for epithelium thickness and *L*_*P*_*b*__ for basal cell layer thickness. No granular layer is present in DSP, therefore there is no distinction between whole epithelium and basal-spinous epithelium thickness. Function *g*_*B*_ (red line, right hand side) takes values of approximately 1 for basal cells, dropping to approximately zero everywhere else. Function *g*_*G*_ (black dashed line) is approximately 1 for the basal-spinous epithelium, dropping to approximately zero for granular cells.

A mathematical model was developed to investigate the potential determinants of FMDV lysis. The model is aimed at investigating the spread, cell infiltration and cell lysis by virions introduced into epithelial tissue. As events occur over space and time, the model is formulated in terms of a system of linked nonlinear partial differential equations (PDEs). For simplicity, the model describes the dynamics of FMDV in a column of epithelium, so that there is only one spatial dimension (Fig 3). Moreover, the model only considers the dynamics of FMDV in epithelium over the short-term (approximately 48 hours), for a timescale sufficient for lesions to occur but before the adaptive immune response begins to play a significant role. Model variables are presented in Table 1. Taking an Occam’s razor approach, it is assumed that cells in the DSP and tongue are fundamentally the same, with the exception of already described structural differences between the two tissues.

**Table 1:** Model variables.

	**Variable**	**Units**
*S*_*c*_	Cellular volume fraction	(non dimensional)
*S*_*e*_	Extracellular volume fraction	(non dimensional)
*V*_*c*_	Cellular viral concentration	PFU × cm^−1^
*V*_*e*_	Extracellular viral concentration	PFU × cm^−1^
*K*	Intracellular resource	cm^−1^
*E*	Activator concentration	cm^−1^
*v*	Velocity of cells	cm × h^−1^
*u*	Velocity of extracellular fluid	cm × h^−1^

#### Model structure

The PDE model of FMDV dynamics in epithelial tissue considers both the tissue fraction of epithelial cells and extracellular space (*S*_*c*_ and *S*_*e*_ respectively) and the viral concentration in them (*V*_*c*_ and *V*_*e*_ respectively).

To allow for the lytic effect of FMDV replication the model also includes the amount of an intracellular resource (*K*). The intracellular resource is a general term representing the resources which the virus exploits for its replication but are essential for cell function and survival. Depletion of the intracellular resource *K* due to viral replication leads to cell death in the model. Given the lack of information on mechanism(s) involved in FMDV-induced cell death [10], this study focuses on the events of cell lysis in general, rather than distinguishing between apoptosis and necrosis as the type of cell death preceding it.

Epithelial structure is incorporated in the model using an activator. This is a general term representing the combination of resources (e.g. nutrients, chemical signals) which account collectively for the ability of cells to proliferate and differentiate into different types within the epithelium. It is assumed the activator diffuses through the epithelium after being delivered at the basement membrane and that different cell layers are defined by the level of activator available to them; cell layer boundaries are located where the concentration of activator, *E*, drops below certain thresholds. To inform the activator parameters of this model, data on epithelial growth factor (EGF) were used (see S3 Supplementary Information).

The distance from the basement membrane, *x*, is measured in cm and the time, *t*, is measured in hours.

#### Response functions

Epithelium cell layer structure is incorporated in the model by several functions of the activator *E*. Cell proliferation occurs in the basal layer [11], while viral replication and uptake are believed to be restricted to basal and spinous cells. The latter stems from the recorded presence of FMDV and of the integrins involved with FMDV uptake in basal and spinous layers [9, 12] and the absence of such data for the granular layer. Cell layers are defined by continuous functions which express more accurately the continuous nature of the epithelium and the lack of clear boundaries between layers. Function *g*_*B*_(*E*) defines the boundary between the basal and spinous layers, while *g*_*G*_(*E*) defines the boundary between the spinous and granular layers. In this case,

$$\begin{array}{c} g_{B}\left(E\right)=\frac{E^{m_{2}}\left(E_{0}^{m_{2}}+E_{B}^{m_{2}}\right)}{E_{0}^{m_{2}}\left(E^{m_{2}}+E_{B}^{m_{2}}\right)} \end{array}$$ (1)

$$\begin{array}{c} g_{G}\left(E\right)=\frac{E^{m_{3}}\left(E_{0}^{m_{3}}+E_{G}^{m_{3}}\right)}{E_{0}^{m_{3}}\left(E^{m_{3}}+E_{G}^{m_{3}}\right)} \end{array}$$ (2)

where *E*_*B*_ and *E*_*G*_ are the threshold concentration for the basal-spinous boundary and the spinous-granular (tongue) or spinous-epithelium surface (DSP), respectively, while exponents *m*_2_ and *m*_3_ accommodate a sharp transition between layers. A schematic diagram of the values of *g*_*B*_ and *g*_*G*_ over epithelial cell layers is included in Fig 3.

Potential differences in viral replication and uptake between basal and spinous cells are described in the model through the use of parameters indicating cell permissiveness to viral replication, *ρ*_*B*_ and *ρ*_*S*_, and cell vulnerability to viral uptake, *μ*_*B*_ and *μ*_*S*_. Preference of one layer over the other is expressed by higher values for the parameters referring to that layer. For example, higher FMDV replication in the basal layer means that *ρ*_*B*_ > *ρ*_*S*_. The absolute difference of the vulnerability parameters in each case, ∣*ρ*_*B*_−*ρ*_*S*_∣ and ∣*μ*_*B*_−*μ*_*S*_∣, is a measure of the difference in the viral preference between cell layers. These differences are incorporated in the model by the functions *h*_*R*_(*E*) and *h*_*U*_(*E*) given by,

$$\begin{array}{c} h_{R}\left(E\right)=\rho_{S}+\left(\rho_{B}-\rho_{S}\right)g_{B}\left(E\right) \end{array}$$ (3)

$$\begin{array}{c} h_{U}\left(E\right)=\mu_{S}+\left(\mu_{B}-\mu_{S}\right)g_{B}\left(E\right) \end{array}$$ (4)

so that *h*_*R*_(*E*) ≃ *ρ*_*S*_ in the spinous layer and *h*_*R*_(*E*) ≃ *ρ*_*B*_ in the basal layer, and likewise for *h*_*U*_(*E*). As there is little experimental evidence of such differences between layers (see S3 Supplementary Information for details), the default values for these parameters are *ρ*_*S*_ = *ρ*_*B*_ = 1 and *μ*_*S*_ = *μ*_*B*_ = 1, but the effect of hypothetical differences will be explored to see if they could be a determinant of lysis. In the absence of experimental data supporting additional differences between epithelial cell layers, and for simplicity, no further differences were incorporated in the model.

The cell death response function *f*(*K*) was assumed to be a Hill like function, such that,

$$\begin{array}{c} f\left(K\right)=\frac{{K_{1/2}}^{m_{1}}}{{K_{1/2}}^{m_{1}}+K^{m_{1}}} \end{array}$$ (5)

where *f*(*K*_1/2_) = 1/2. Depletion of cell resources below this threshold (*K* ≪ *K*_1/2_) triggers cell death at a maximum rate (corresponding to *f*(*K*) ≈ 1).

#### Model equations

Epithelial tissue is assumed to consist only of cellular and extracellular space, so that

$$\underset{\text{extracellular}\;\text{space}\;\text{fraction}}{\underset{︸}{S_{e}\left(x,t\right)}}+\underset{\text{cellular}\;\text{space}\;\text{fraction}}{\underset{︸}{S_{c}\left(x,t\right)}}=1.$$ (6)

In order for cells to proliferate in the basal layer, neighbouring cells need to migrate to accommodate new cell volume. Furthermore, extracellular fluid is drawn in to fill the volume gaps created by cell migration. The rate of such movements is described by the velocity *v* for the cells and *u* for the extracellular fluid. Cell division is governed by the level of activator *E*, and occurs at a rate *βg*_*B*_(*E*). Cell death occurs at a rate dependent on the generic resource *K*, namely Φ*f*(*K*)*S*_*c*_, where Φ is the maximum observed cell lysis rate and *f*(*K*) is a normalised response function. The equations for *S*_*c*_ and *S*_*e*_ are thus

$$\begin{array}{c} \frac{\partial }{\partial t}\left(S_{c}\right)+\frac{\partial }{\partial x}\left(vS_{c}\right)=\underset{\text{FMDV-induced}\;\text{cell}\;\text{death}}{\underset{︸}{-\Phi f(K)S_{c}}}+\underset{\text{cell}\;\text{division}}{\underset{︸}{\beta g_{B}\left(E\right)S_{c}}} \end{array}$$ (7)

$$\begin{array}{c} \frac{\partial }{\partial t}\left(S_{e}\right)+\frac{\partial }{\partial x}\left(uS_{e}\right)=\underset{\text{FMDV-induced}\;\text{cell}\;\text{death}}{\underset{︸}{\Phi f(K)S_{c}}}-\underset{\text{cell}\;\text{division}}{\underset{︸}{\beta g_{B}\left(E\right)S_{c}}}. \end{array}$$ (8)

The activator is delivered through the basement membrane and diffuses through the epithelium (with diffusion constant *D*_*E*_). Living cells take up activator at rate *λ*, while there is also natural decay (at rate *δ*). The activator dynamics are described by

$$\frac{\partial E}{\partial t}=\underset{\text{activator}\;\text{diffusion}}{\underset{︸}{D_{E}\frac{\partial^{2}E}{\partial x^{2}}}}-\underset{\text{activator}\;\text{uptake}\;\text{by}\;\text{cells}}{\underset{︸}{\lambda S_{c}E}}-\underset{\text{activator}\;\text{decay}}{\underset{︸}{\delta E}}.$$ (9)

Since the activator diffuses much more rapidly than FMDV (see Table 2), it is assumed that its distribution is in a near equilibrium (or quasi-steady) state over the timescale of viral dynamics. Using this assumption we obtain

$$D_{E}\frac{\partial^{2}E}{\partial x^{2}}=\lambda S_{c}E+\delta E.$$ (10)

**Table 2:** Model parameters, their interpretation and ‘standard’ values used in the simulations.

	**Parameter**	**Value**
*β*	maximum rate of cell proliferation	1.33 × 10^−2^ h^−1^ [13]
*λ*	uptake rate of activator by cells	1.97 × 10^−12^ h^−1^ [14]
*δ*	decay rate of activator	0.693 h^−1^ [15]
*D*_*E*_	diffusion coefficient of activator	1.86 × 10^−3^ cm^2^ h^−1^ [16]
Φ	maximum rate of cell lysis due to viral infection	3.33 × 10^−1^ h^−1^ [17]
*ξ*	maximal replication rate of virus	1.56 × 10^−1^ PFU /resource fraction (based on [24])
*ρ*	rate at which virus uses up intracellular resource	2.46 × 10^−2^/(PFU × h × cm^−1^ (based on [24])
*μ*	virion-cell affinity and internalisation rate	2 × 10^−5^ h^−1^ (based on [18])
*γ*	rate of virus release by live cells	0 h^−1^ (see S3 Supplementary Information)
*D*_*V*_	diffusion coefficient of virions	3.67 × 10^−4^ cm^2^/ h [19]
*ρ*_*B*_	defines relative susceptibility of basal layer to FMDV replication	1 (see S3 Supplementary Information)
*ρ*_*S*_	defines relative susceptibility of spinous layer to FMDV replication	1 see S3 Supplementary Information)
*μ*_*B*_	defines relative vulnerability of basal layer to FMDV infection	1 (see S3 Supplementary Information)
*μ*_*S*_	defines relative vulnerability of spinous layer to FMDV infection	1 (see S3 Supplementary Information)
*K*_1/2_	value of K at which cell death is half maximum value	38.1 resource units × cm^−1^ (see S3 Supplementary Information)
*m*_1_	exponent in function *f* which defines resource depletion	4 (see S3 Supplementary Information)
*m*_2_	exponent in function *g*_*B*_ which defines the basal layer	80 (see S3 Supplementary Information)
*m*_3_	exponent in function *g*_*G*_ which defines the basal and spinous layers	80 (see S3 Supplementary Information)
*L*_*P*_	thickness of dorsal soft palate	1.71 × 10^−2^ cm (measured data)
*L*_*T*_	thickness of tongue	1.66 × 10^−1^ cm (measured data)
*L*_*Pb*_	threshold of spinous cell layer in dorsal soft palate	1.41 × 10^−3^ cm (measured data)
*L*_*Tb*_	threshold of spinous cell layer in tongue	1.22 × 10^−3^ cm (measured data)
*L*_*Tg*_	threshold of granular cell layer in tongue	1.59 × 10^−1^ cm (measured data)
*E*_*B*_	concentration of *E* at basal-spinous interface	9.66 × 10^−1^ (DSP) (based on measured data), 9.77 × 10^−1^ (tongue) (based on measured data)
*E*_*G*_	concentration of *E* at spinous-granular interface (DSP) or spinous-surface interface (tongue)	6.3 × 10^−1^ (DSP) (based on measured data), 8.18 × 10^−2^ (tongue) (based on measured data)

The values are either taken from the indicated source, measured as part of the study or estimated (see S3 Supplementary Information).

Viral uptake, as well as replication, depends on the cell type, with inter-layer variability described by functions *g*_*G*_(*E*) and *h*_*U*_(*E*) for uptake and *g*_*G*_(*E*) and *h*_*R*_(*E*) for replication. Replication of FMDV occurs within cells, at a maximal rate *ξ*, resulting in the consumption of the generic resource, *K*, at an hourly rate *ρ* per unit of virus concentration. Uptake of extracellular virus by cells (infection), at a rate *μ*, is enabled by the presence of receptors on the cell surface. Release of FMDV into the extracellular space can occur by two processes: (non-lytic) escape from infected cells while alive (at rate *γ*); and release of virus during lysis. No direct cell-to-cell virus movement is assumed to occur, so virus must pass through the extracellular space first. In the intracellular space viral particles are assumed to be transported only by cell movement. In the extracellular space, however, viral particles are assumed to be transported via diffusion. The equations of *V*_*c*_ and *V*_*e*_ are thus,

$$\begin{array}{cc} \frac{\partial }{\partial t}\left(V_{c}S_{c}\right)+\frac{\partial }{\partial x}\left(vV_{c}S_{c}\right)= & \underset{\text{FMDV}\;\;\text{replication}}{\underset{︸}{\xi \rho Kh_{R}\left(E\right)g_{G}\left(E\right)V_{c}S_{c}}}+\underset{\text{FMDV}\;\text{uptake}\;\text{by}\;\text{cells}}{\underset{︸}{\mu g_{G}\left(E\right)h_{U}\left(E\right)V_{e}S_{c}}}\\ & -\underset{\text{FMDV}\;\text{release}\;\text{by}\;\text{live}\;\text{cells}}{\underset{︸}{\gamma V_{c}S_{c}}}\\ & -\underset{\text{FMDV}\;\text{release}\;\text{due}\;\text{to}\;\text{FMDV-induced}\;\text{cell}\;\text{lysis}}{\underset{︸}{\Phi f\left(K\right)V_{c}S_{c}}} \end{array}$$ (11)

$$\begin{array}{cc} \frac{\partial }{\partial t}\left(V_{e}S_{e}\right)+\frac{\partial }{\partial x}\left(uV_{e}S_{e}\right)= & -\underset{\text{FMDV}\;\text{uptake}\;\text{by}\;\text{cells}}{\underset{︸}{\mu g_{G}\left(E\right)h_{U}\left(E\right)V_{e}S_{c}}}+\underset{\text{FMDV}\;\text{release}\;\text{by}\;\text{live}\;\text{cells}}{\underset{︸}{\gamma V_{c}S_{c}}}\\ & +\underset{\text{FMDV}\;\text{release}\;\text{due}\;\text{to}\;\text{FMDV-induced}\;\text{cell}\;\text{lysis}}{\underset{︸}{\Phi f\left(K\right)V_{c}S_{c}}}\\ & +\underset{\text{FMDV}\;\text{diffusion}}{\underset{︸}{D_{V}\frac{\partial }{\partial x}\left(S_{e}\frac{\partial V_{e}}{\partial x}\right)}}. \end{array}$$ (12)

Intracellular resource, *K*, is assumed to be contained within cells and to move within space as a result of cell migration only. As basal cells grow and divide, a corresponding amount of resource is produced (at rate *βg*_*B*_(*E*)*KS*_*c*_). This amount of resource is maintained by healthy cells but is depleted following infection at a rate proportional to viral replication (rate *ρKh*_*R*_(*E*)*g*_*G*_(*E*)*V*_*c*_ *S*_*c*_). The equation for the intracellular resource, *K*, is thus

$$\begin{array}{cc} \frac{\partial }{\partial t}\left(KS_{c}\right)+\frac{\partial }{\partial x}\left(vKS_{c}\right)= & -\underset{\text{loss}\;\text{of}\;\text{resource}\;\text{due}\;\text{to}\;\text{FMDV}\;\text{replication}}{\underset{︸}{\rho Kh_{R}\left(E\right)g_{G}\left(E\right)V_{c}S_{c}}}\\ & -\underset{\text{loss}\;\text{of}\;\text{resource}\;\text{due}\;\text{to}\;\text{FMDV-induced}\;\text{cell}\;\text{lysis}}{\underset{︸}{\Phi f\left(K\right)KS_{c}}}\\ & +\underset{\text{production}\;\text{of}\;\text{resource}\;\text{due}\;\text{to}\;\text{cell}\;\text{division}}{\underset{︸}{\beta g_{B}\left(E\right)KS_{c}}} \end{array}$$ (13)

where Φ*f*(*K*)*KS*_*c*_ is the rate of resource loss in the system due to cell death.

The model parameters are summarised in Tables 2 and 3. Parameter estimates were obtained from the published literature (references [13–17, 19, 20]) or, in the case of epithelium structure, were derived from direct measurement. Full details are provided in S3 Supplementary Information (where references [21–24] provide additional information on parameter estimates). For the estimation of parameters *ξ* and *ρ*, a simplified, non-spatial form of the dimensional model was used in combination with relevant in vitro data (see S4 Supplementary Information and reference [18]). The parameter estimates in Tables 2 and 3 were used as a starting point for the exploration of the system, with global sensitivity analysis complementing its investigation.

**Table 3:** Initial and boundary conditions parameters.

	**Parameter**	**Condition**	**Value**
*α*	initial cellular space volume	*S*_*c*_(*x*, 0)	0.95 [8]
*E*_0_	activator at the basement membrane	*E*(0, *t*)	1 cm^−1^
*K*_0_	initial intracellular resource fraction per unit length	*K*(*x*, 0)	952 cm^−1^
*V*_0_	viral infectious dose	*V*_*e*_(*e*_*p*_, 0)	2290 PFU × cm^−1^
*Q*_*E*_	activator mass transfer coefficient	$\frac{\partial E}{\partial x}\left(L_{P}\right)$	5.69 × 10^−2^ cm × h^−1^
*Q*_*V*_	FMDV mass transfer coefficient	$\frac{\partial V_{e}}{\partial x}\left(L_{P}\right)$	2.85 × 10^−2^ cm × h^−1^
*e*_*p*_	viral entry point	various points tested	0, 3 × 10^−3^cm, *L*_*i*_ or *L*_*i*_−3 × 10^−3^ cm, where *i* = *P*,*T*

See S3 Supplementary Information for details.

#### Boundary and initial conditions

Initially, the epithelium is assumed to be healthy and intact, so that the cellular volume fraction, *S*_*c*_, and the intracellular resource, *K*, are at a maximum healthy level, *α* and *K*_0_ respectively. Viral concentration is equal to zero everywhere except at the point of infection, *e*_*p*_, which is assumed to be a point in the extracellular space. Details on the selection of four potential viral entry points are given in S3 Supplementary Information. The basement membrane is considered to be the most likely entry point for the tongue and the epithelium surface for the DSP. The initial conditions are thus,

$$t=0:\;S_{c}=\alpha ,\;S_{e}=1-\alpha ,\;K=K_{0},\;V_{c}=0,\;V_{e}\left(x,0\right)=V_{0}\delta \left(x-e_{p}\right)$$

where *δ*(.) is Dirac’s delta function.

The activator, *E*, diffuses from the basement membrane, at *x* = 0, and is assumed to be at a fixed concentration there. Extracellular virus, *V*_*e*_, is allowed to diffuse out of the basement membrane depending on a mass transfer coefficient, *Q*_*V*_.

$$E\left(0,t\right)=E_{0},\;-D_{V}\frac{\partial V_{e}}{\partial x}\left(0,t\right)=-Q_{V}V_{e}\left(0,t\right)$$

One of the main differences between the DSP and the tongue is the presence of the keratinised corneal layer in the tongue which acts as a barrier to the passage of activator and extracellular virus. For the tongue it is assumed,

$$\frac{\partial E}{\partial x}\left(L_{T},t\right)=0,\;\frac{\partial V_{e}}{\partial x}\left(L_{T},t\right)=0.$$

In the DSP there is no such barrier and Robin conditions are imposed on the boundary, namely,

$$-D_{E}\frac{\partial E}{\partial x}\left(L_{P},t\right)=Q_{E}E\left(L_{P},t\right),\;-D_{V}\frac{\partial V_{e}}{\partial x}\left(L_{P},t\right)=Q_{V}V_{e}\left(L_{P},t\right).$$

Activator and virus obey Fick’s law of mass transfer and move from an area of high concentration (epithelium) to an area of low concentration (outside the epithelium) in line with mass transfer coefficients, *Q*_*E*_ and *Q*_*V*_ respectively.

Using the boundary conditions of activator, *E*, and assuming that the cellular volume fraction, *S*_*c*_, is constant (*S*_*c*_ = *α*), Eq (10) is solved to obtain the initial condition for activator concentration

$$\begin{array}{cc} E(x,0)= & \frac{E_{0}\left(A-\frac{Q_{E}}{D_{E}}\right)e^{-2AL_{P}}e^{Ax}}{\frac{Q_{E}}{D_{E}}\left(1-e^{2AL_{P}}\right)+A\left(1+e^{2AL_{P}}\right)}\\ & +\frac{E_{0}\left(\frac{Q_{E}}{D_{E}}+A\right)e^{-Ax}}{\frac{Q_{E}}{D_{E}}\left(1-e^{-2AL_{P}}\right)+A\left(1+e^{-2AL_{P}}\right)},\;\;0\leq x\leq L_{P}\;\text{in}\;\text{palate}, \end{array}$$ (14)

$$\begin{array}{c} E\left(x,0\right)=\frac{E_{0}e^{Ax}}{1+e^{2AL_{T}}}+\frac{E_{0}e^{2AL_{T}}e^{-Ax}}{1+e^{2AL_{T}}},\;\;0\leq x\leq L_{T}\;\text{in}\;\text{tongue}, \end{array}$$ (15)

where $A=\sqrt{\left(\lambda \alpha +\delta \right)/D_{N}}$. This is the state of the system in the absence of virus.

### Numerical methods

To make implementation of the model more efficient the Eqs (6)–(13) were non-dimensionalised. Moreover, in the non-dimensional equations cell proliferation was negligible over the time-scale of the viral dynamics and so could be ignored (see S1 Supplementary Information). Spatial derivates were replaced with their central difference approximations and the resulting ordinary differential equations were solved numerically in Matlab [25] using a variable order solver for stiff systems (based on Gear’s method).

### Sensitivity analysis

Latin hypercube sampling (LHS) [26] was used to explore the sensitivity of the model predictions to changes in the parameter estimates. A hundred replicates were conducted for each sensitivity analysis, this number being well above the recommended lower limit of LHS replicates [27]. Parameter values were chosen logarithmically over the chosen range, with the exception of values for exponents *m*_1_, *m*_2_, *m*_3_ which were chosen linearly over the range [1, 100].

## Results

### Investigation of the role of epithelial tissue structure in FMDV-induced lysis

**Fig 4:**
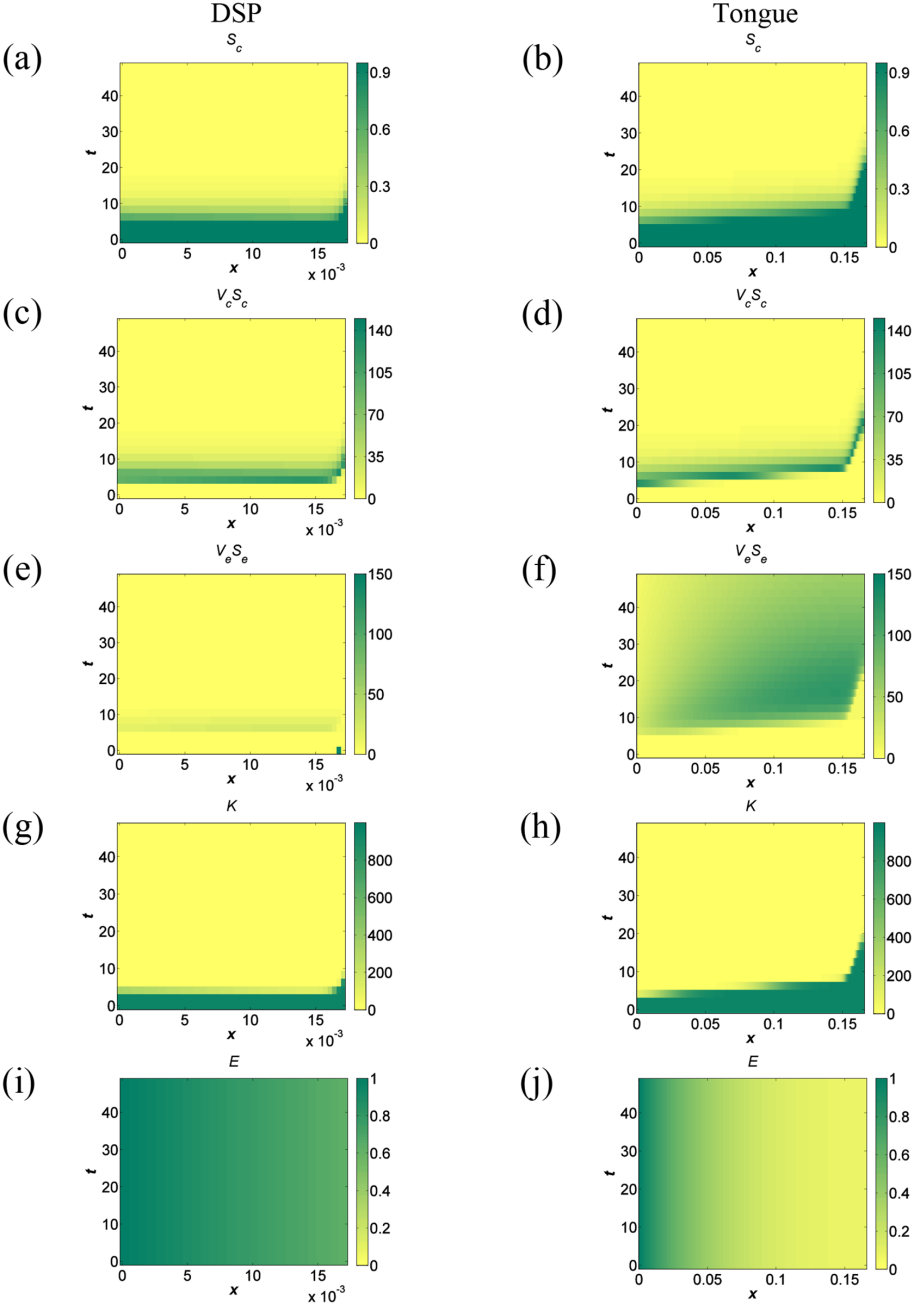
Simulation results for DSP and tongue over a 48 hour timescale. Epithelium surface used as the viral entry point for DSP and basement membrane as the viral entry point for tongue. (a), (b) Cellular space fraction, *S*_*c*_, of DSP and tongue respectively. (c), (d) Intracellular viral load, *V*_*c*_ *S*_*c*_, of DSP and tongue respectively measured in PFU/cm. (e), (f) Extracellular viral load, *V*_*e*_ *S*_*e*_, of DSP and tongue respectively measured in PFU/cm. Green area at the bottom right hand corner of (e) indicates viral entry in DSP. Due to different scaling of (f), viral entry in tongue is not visible (bottom left hand corner). (g), (h) Intracellular resource, *K*, of DSP and tongue respectively, measured in cm^−1^. (i), (j) Activator concentration, *E*, of DSP and tongue respectively.

The model was investigated numerically for both DSP and tongue using the default parameter values (Table 2). All four entry points tested (see Table 3) had similar effects on the behaviour of the system: fast and complete destruction of the intracellular resource, *K*, and of the cellular fraction, *S*_*c*_, and the presence of intracellular virus, *V*_*c*_, and extracellular virus, *V*_*e*_, in the tissue. In Fig 4 results of the cases corresponding to the most likely source of infection for each tissue are presented; DSP is a site of primary infection with FMDV [5] thus *e*_*p*_ = *L*_*P*_, and tongue is usually infected through viraemia [5] so here *e*_*p*_ = 0. These results are independent of the differences in epithelial tissue thickness with no signs of surviving cellular fraction when tongue thickness, *L*_*T*_, is reduced to the thickness of DSP, or DSP thickness, *L*_*P*_, increased to the size of tongue.

An initial exploration of the system indicated that the most influential parameters are the viral replication parameters *ξ* and *ρ*, but parameters *K*_1/2_, *μ*, *D*_*V*_, *Q*_*V*_, *V*_0_, *m*_1_, *m*_2_, *m*_3_, *ρ*_*B*_, *ρ*_*S*_, *μ*_*B*_ and *μ*_*S*_ were also shown to affect the model dynamics. Of these, parameters *ρ*_*B*_, *ρ*_*S*_, *μ*_*B*_ and *μ*_*S*_ were initially treated as constant assuming no differences in FMDV uptake and replication rates between layers. Sensitivity analyses were carried out for each viral entry point.

**Fig 5:**
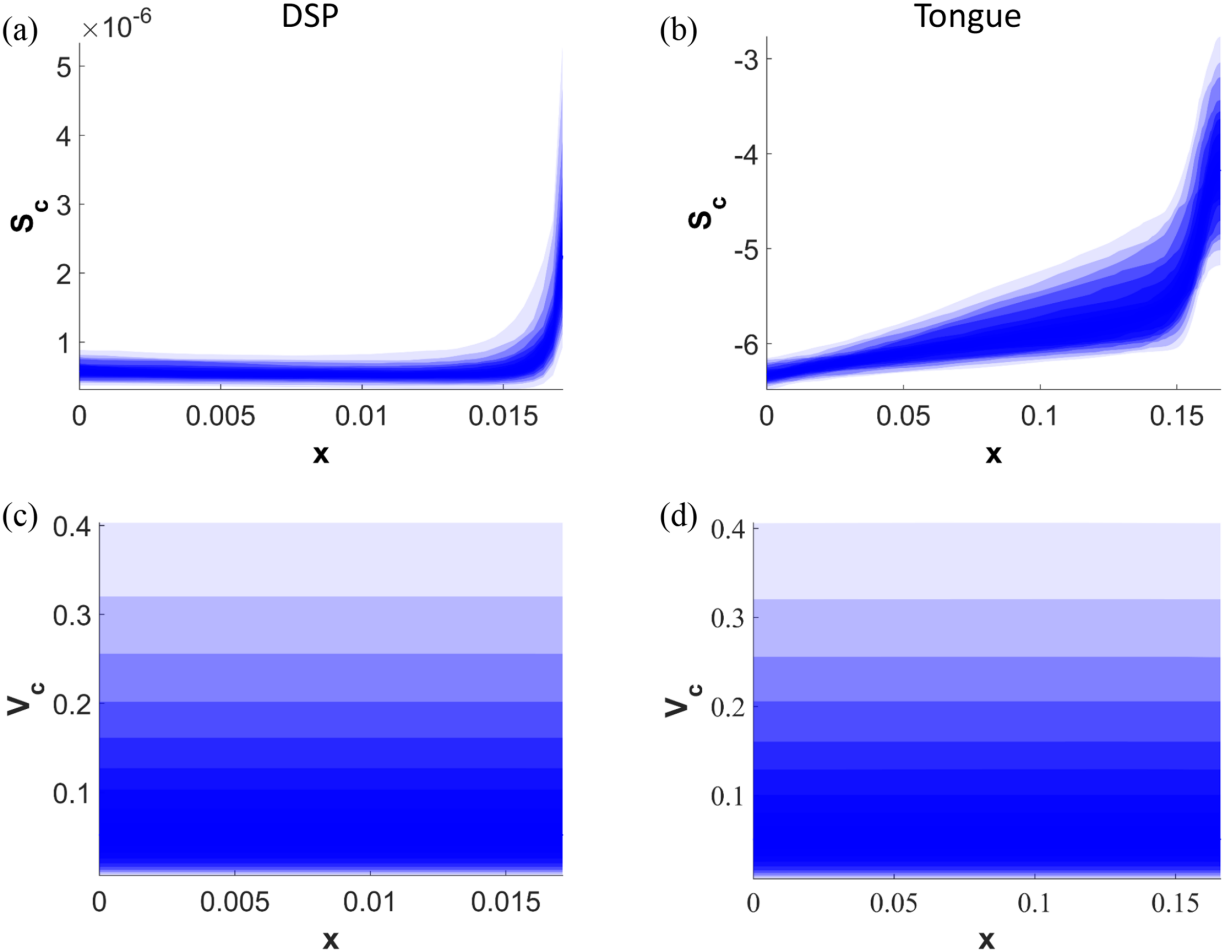
Extensive sensitivity analysis. LHS applied to the model with tested parameters ranging from 0.1 to 10 times their estimated values shows the consistency of results in predicting destruction of the cellular column. As parameters are varied, *S*_*c*_ in graph (a) of the DSP remains bounded below 1 × 10^−6^ and *V*_*c*_ *S*_*c*_ in graph (c) below 4 × 10^−7^ PFU/cm, apart from at surface where *S*_*c*_ < 6 × 10^−6^ and *V*_*c*_ *S*_*c*_ < 15 × 10^−7^ PFU/cm. In graph (b) of the tongue, *S*_*c*_ is bounded below 10^−5^ for most of the tissue, but closer to the granular layer *S*_*c*_ < 3 × 10^−3^. Similarly, in graph (d) *V*_*c*_ *S*_*c*_ is bounded below 10^−5^ PFU/cm for most of the tissue, but closer to the granular layer *V*_*c*_ *S*_*c*_ < 5 × 10^−4^ PFU/cm. The range of possible results is plotted in 5 percentile steps (shaded), from 100 replicates. Parameters tested: *K*_1/2_, *μ*, *D*_*V*_, *Q*_*V*_, *V*_0_, *m*_1_, *m*_2_, *m*_3_.

In Fig 5 results of the LHS sensitivity analysis for the default viral entry points for DSP and tongue are presented. Because the model is highly sensitive to parameters *ξ* and *ρ*, these are excluded from this investigation, but their effect on the model is explored more extensively in the next section. Parameters explored here are *K*_1/2_, *μ*, *D*_*V*_, *Q*_*V*_ and *V*_0_ for which tested values range from 0.1 × to 10 × their default estimates, and *m*_1_, *m*_2_, and *m*_3_ which take values from the range [1, 100]. Results presented in Fig 5 are consistent with the findings of the model for the original parameter values, predicting cell death of the entire epithelial cell column. Other viral entry points produce similar results.

### Sensitivity of the model to viral replication parameters

The maximal replication rate of FMDV, *ξ*, and the rate of FMDV resource consumption, *ρ*, were highlighted as the parameters to which the model is most sensitive. A reduction of the maximal replication rate to 7.5% of its default estimate or a decrease of the FMDV resource consumption rate to 9% of its default estimate result in a substantial difference in the surviving cellular space fraction between tongue and DSP (88% in DSP vs 77% in tongue and 89% in DSP vs 80% in tongue, respectively). A simultaneous alteration to the values of both parameters at 29% of their default values has a similar outcome (79% in DSP vs 67% in tongue). These modifications in the default values of these parameters are the minimum required to achieve a difference in the level of cell survival between the DSP and tongue. We note that simulation results will still show destruction of cells in both tissues if allowed to run for longer times, but this occurs at a timescale beyond the scope of the current model.

**Fig 6:**
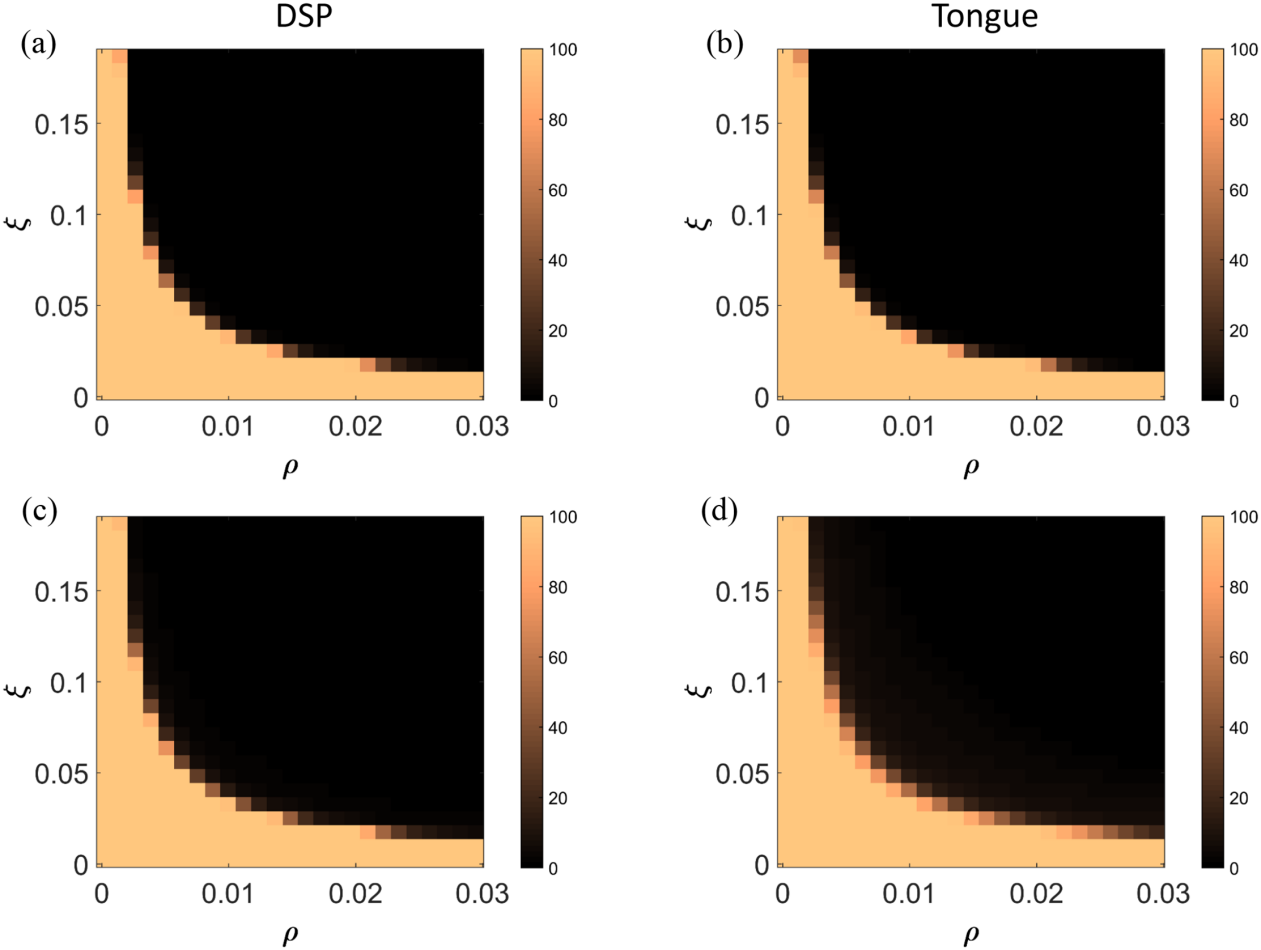
Sensitivity analysis for alterations to viral replication parameter estimates. Model sensitivity to alterations of the estimates of the maximal replication rate of FMDV, *ξ*, and the rate of intracellular resource consumption by FMDV, *ρ*, as exhibited by the percentage of the remaining cellular space fraction, *S*_*c*_, 48 hours post infection. (a), (b) Percentage of minimum surviving cellular space fraction over the whole tissue column for the case of DSP and tongue respectively. (c), (d) Percentage of average surviving cellular space fraction over the whole tissue column for the case of DSP and tongue respectively.

Further exploration of the system with respect to the combined effect of these two parameters is presented in Fig 6. Values of the two parameters for which there is a different behaviour of the system between the two tissues in regards to the levels of cell survival can be identified, but the results are highly sensitive to small changes to these values.

### Effect of the level of viral uptake by cells and viral diffusion on cell death

The uptake rate of FMDV by cells, *μ*, and the diffusion rate of FMDV, *D*_*V*_, did not greatly influence the model predictions, whether varied in combination or individually. This outcome for parameter *μ* is particularly unexpected as the uptake rate of virus by cells and, therefore, the receptor distribution, are generally considered of major importance for the infection of cells by FMDV and have been suggested as potential determinants for epithelial cell death [28, 29]. Total destruction of both tissues is observed for *μ* as low as 10^−20^ × its default value. Changes to the value of *D*_*V*_ of up to three orders of magnitude result in total or large scale destruction of the cellular space.

### Role of potential differences in viral uptake and replication between cell layers

As described above, the model shows that based on the current estimates of the viral replication parameters *ξ* and *ρ*, and for any level of viral uptake, *μ*, (aside of *μ* = 0) there is complete destruction of the whole cellular column. Consequently, any differences in viral replication and/or viral uptake between basal and spinous layers will not affect the occurrence of extensive cell death as long as the default estimates for these parameters reflect the dynamics of the system in at least one layer. It is noted here that extensive cell death in FMDV-infected epithelial tissues is considered to lead to the formation of vesicular lesions regardless of the epithelium depth where it occurs.

Nevertheless we set out to investigate the effect of the potentially different vulnerability of basal and spinous layers to infection and viral replication for parameter estimates which allow the survival of cells. For this reason parameters *ξ* and *ρ* were set to the previously identified level of 29% of their default estimates. Results show the difference in the minimum cell survival in the two epithelia to widen when basal cells are more prone to FMDV infection or replication (see Figs S5.1-S5.3 in S5 Supplementary Information). Interestingly, it has been suggested that integrin αvβ6, which is thought to act as the main FMDV receptor [9], is expressed more in spinous than basal cells [9] making the extraction of conclusions even more complicated. Based on the model results though, it appears that different FMDV replication rates between different cell layers and different receptor distribution between different cell layers could cause a substantial difference in the levels of cell survival between DSP and tongue.

### Effect of other parameters in combination with viral replication parameters

A difference in the levels of cell survival between DSP and tongue were observed for a 40% increase in the estimate of the mass transfer coefficient of FMDV, *Q*_*V*_, or a 25% decrease in the estimate of the threshold amount of resource for cell death, *K*_1/2_ (see Figs S5.4 & S5.5 in S5 Supplementary Information).

### Role of the site of infection

**Fig 7:**
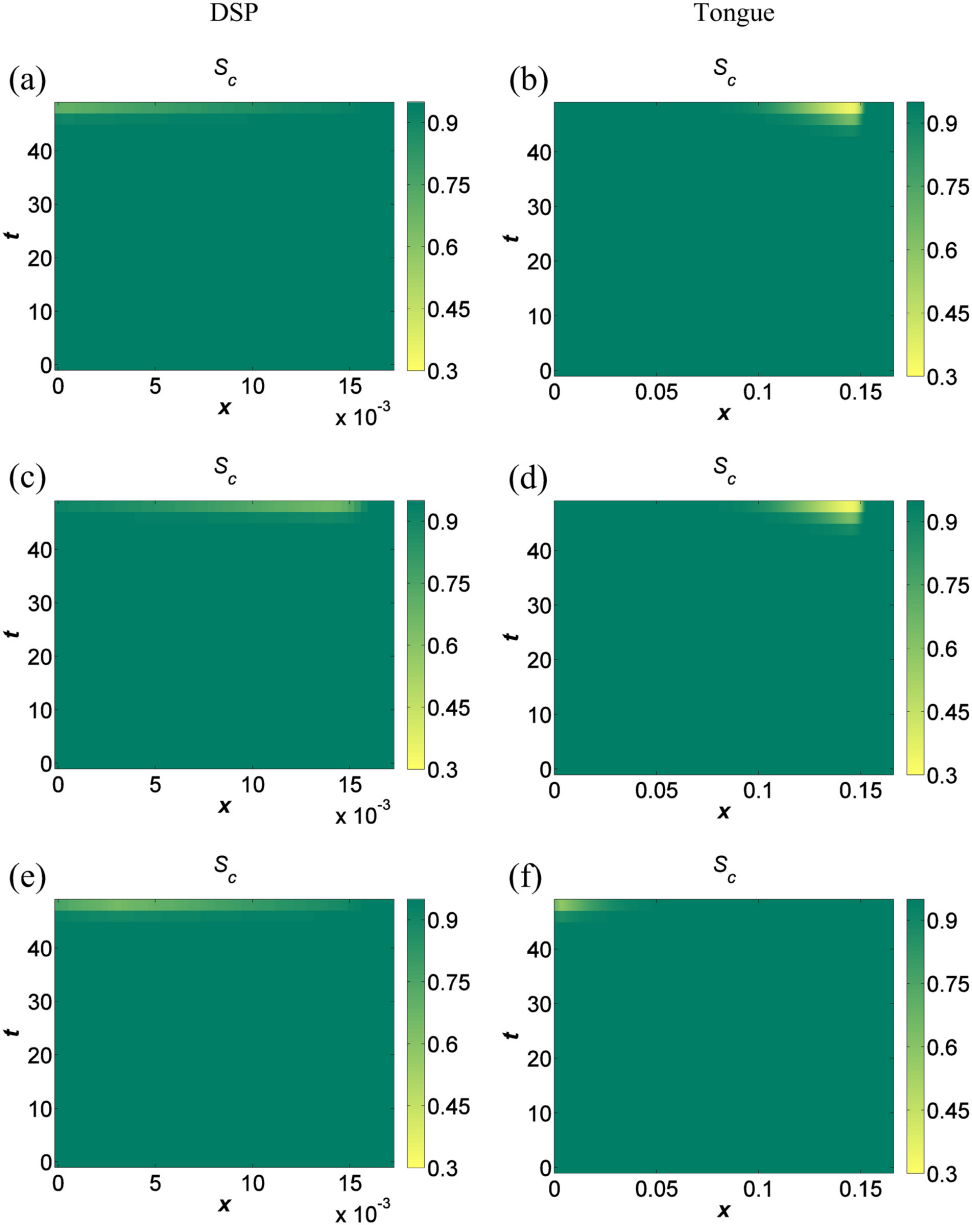
Simulation results for different viral entry points. Simulation results of cellular space fraction, *S*_*c*_, for DSP and tongue over a 48 hour timescale for maximal replication rate, *ξ*, and rate of FMDV resource consumption, *ρ*, both set at 0.29 × their default estimates. (a), (b) Basement membrane used as the viral entry point for DSP and epithelium surface as the viral entry point for tongue respectively. (c), (d) Viral entry point for both DSP and tongue set at *L*_*i*_−3 × 10^−3^ cm. (e), (f) Viral entry point for both DSP and tongue set at 3 × 10^−3^ cm.

Setting the level of the maximal replication rate, *ξ*, and the level of resource consumption by FMDV, *ρ*, at 0.29 × their default estimates, we explored the effect of the site of viral entry to the epithelium (Fig 7). DSP exhibits higher levels of cell survival than tongue for all cases. Comparing the lowest level of cell survival for DSP, which is the result of viral entry at a point at about two cells distance from the basement membrane, with the highest level of tongue survival which is a result of viral entry at the basement membrane, there is still more cell survival in the DSP. For both tissues, higher levels of survival are observed for the default viral entry points, while interestingly the lowest levels of cell survival for the tongue are exhibited when FMDV enters on the granular layer surface or about two cells in from there.

### Effect of other parameters

In the investigation of the model only two more parameters had an effect on the levels of cell survival. Results showed that changes in the rate of FMDV release by live cells, *γ*, and the maximum rate of cell death, Φ, can limit the destruction of cellular space, but their effect is similar in both DSP and tongue.

## Discussion

In this study we have used mathematical modelling to explore a range of potential mechanisms which could explain why lesions form in certain epithelial tissues (e.g. tongue) but not in others (e.g. DSP). Specifically, we considered whether this difference in the outcome of infection could result from differences in: (i) epithelium size and structure; (ii) receptor distribution between tissues; (iii) receptor distribution in different layers of the same tissue; (iv) viral replication rates between tissues; and (v) viral replication rates in different layers of the same tissue. Importantly, these hypotheses would be very difficult to test experimentally.

Based on current parameter estimates, the model predicted cell death of the entire column in both the tongue and DSP. This level of FMDV-induced cell death is unrealistic; experimental observations of vesicular lesions indicate that these are not a result of such excessive cell death. The model results suggest that epithelial tissue thickness and cell layer structure alone are not sufficient to account for the difference in behaviour of the tissues. This is supported by simulations where the thickness of both the tissues was altered to resemble the other, while their cell layer structure is maintained. Furthermore, extensive sensitivity analysis of the system showed the model predictions to be robust to a ten-fold increase or decrease in the parameter estimates, with the exception of viral replication parameters *ξ* and *ρ*.

Changes to the maximal replication rate of FMDV, *ξ* and the rate at which FMDV uses up intracellular resource, *ρ* can produce a different behaviour of the two tissues, with DSP exhibiting higher cell survival than tongue. This result is, however, highly sensitive to small changes to *ξ* and *ρ*. This suggests that while the current viral replication rate estimates cannot drive the different behaviour, differences in these rates between tissues could. This latter possibility is considered unlikely based on the available data for FMDV, since epithelial tissues are fundamentally the same.

Expression levels of integrin αvβ6, differ markedly between tongue and DSP, with consistently high levels of expression in tongue and no detectable expression in DSP [9]. This observation led the authors of the study to suggest this integrin as the major receptor related to FMDV epithelial tropism. In this case receptor distribution could be a determinant of cell death, but only if αvβ6 is the exclusive integrin of FMDV. However, integrins αvβ1 [30], αvβ3 [31] and αvβ8 [32] have also been reported to facilitate FMDV infection. This makes it unlikely αvβ6 to be the only receptor used by FMDV. Indeed, the insensitivity of the model predictions to the uptake rate of FMDV by cells, *μ*, suggest that, although presence of receptors is essential for epithelial cell infection and lysis, a difference in receptor distribution between different epithelial tissues is not a determinant of cell death.

Having explored different levels of vulnerability of basal and spinous layers to infection and viral replication in combination with lower estimates for *ξ* and *ρ*, we can conclude that these differences can intensify the difference in the lytic behaviour between the two tissues. Interestingly, although the model results show a bigger gap in the levels of cell survival between DSP and tongue for the case of reduced FMDV replication and uptake in the spinous layer, integrin αvβ6 has been suggested to be expressed at higher levels in spinous than basal cells [9] making conclusions difficult to draw. Based on the results of this study different FMDV replication rates between different cell layers and different receptor distribution between different cell layers cannot be yet rejected as possible determinants of cell death.

Our study examined FMDV dynamics in a single epithelial cell column with FMDV and activator transport between basement membrane and epithelium surface. In reality, the early stages of infection will affect a few ‘columns’ of cells, a situation which the current model should represent reasonably well. However, activator and virus will also spread outwards to neighbouring cells. Although activator concentration across layers wouldn’t be altered as activator is assumed to be delivered at a constant rate throughout the basement membrane, this would not be the case for FMDV. Viral spread will lead to a wave of infection and lysis expanding from the initial infection site. A 3D, or radially symmetric 2D, model would be needed to capture this and this is an interesting avenue to explore in the future.

Our results about the roles of structure and viral replication are consistent with an earlier, ordinary differential equation model of FMDV infection in epithelium [8]. The model of Schley et al. (2011) predicted the development of lesions in thick epithelial tissues and not in thin epithelia, only when the estimates of the basal cells proliferation rate and the maximum cell death rate were altered. In the present study a proliferation rate of epithelial cells similar to the one used to the aforementioned paper was considered negligible for the timescale of interest and was therefore eliminated from the model. The maximum cell death rate was investigated as a parameter which potentially affects lysis, but our results have shown this not to result in any differences in cell death between tongue and DSP. It should be noted that Schley et al. estimated the average thickness of the live epithelial tissues of tongue and DSP to be considerably lower than the estimates of the authors; live tongue epithelium was estimated to be 2.3 × 10^−2^ cm and DSP epithelium 9 × 10^−3^ cm [8]. In the present study measurements suggest live tongue epithelium to be about 1.66 × 10^−1^ cm and DSP to be 1.71 × 10^−2^ cm. Furthermore, our study enables the investigation of potential differences between epithelial cell layers, while it provides the framework for further hypothesis testing in the future.

Finally, this work highlights the relationship between the timescale of cell infection and that of the intracellular events leading to cell lysis and viral release. Since viral uptake occurs much faster than the chain of events leading to viral release (viral replication—resource consumption—cell lysis), different rates of viral uptake have negligible effect on the system because further cell infection depends on the death rate of already infected cells. This suggests that the investigation of FMDV replication and its potential inhibition is more of interest for the control of FMDV-induced cell death, than the investigation of FMDV uptake and the distribution of FMDV receptors.

## Conclusions

The model presented here predicts extensive cell death in both DSP and tongue for the current set of parameter estimates. Moreover, exploring the sensitivity of the model to changes in the parameters indicates that differences in epithelial structure are unlikely to be driving the difference in behaviour between tongue and DSP. Accordingly, additional biological detail needs to be incorporated in the model. A next step when investigating the dynamics of FMDV in epithelial tissues, would be to incorporate effects of the host immune response, for example, the antiviral action of interferon (IFN) or lymphocytes. The IFN response has been shown experimentally to differ between tongue and DSP [33] and so will influence the dynamics of FMDV in these tissues. It has also been speculated that lymphocytes also have a role in FMDV-induced cell lytic events [6] and investigation of this would be of particular interest. The model presented in this paper provides an ideal framework in which to incorporate this additional biological realism.

## Supporting Information

S1 Supplementary Information Mathematical model. Non-dimensional forms of the full and static-cell models.
(PDF)

S2 Supplementary Information Epithelial thickness and cell layer structure. Details on the bovine epithelial cell data collection and analysis.
(PDF)

S3 Supplementary Information Parameter estimation. Detailed explanation of the parameter estimates derivation.
(PDF)

S4 Supplementary Information Reduced model for the estimation of *ρ* and *ξ*. (PDF)

S5 Supplementary Information Supplementary results. (PDF)

S1 Data Supplement Epithelial thickness and cell layer structure data. (XLSX)
