# Supplementary material for: Using Mathematical Modelling to Explore Hypotheses about the Role of Bovine Epithelium Structure in Foot-And-Mouth Disease Virus-Induced Cell Lysis
Source: PLoS One. 2015 Oct 2;10(10):e0138571. doi: 10.1371/journal.pone.0138571 (PMC4592007; doi:10.1371/journal.pone.0138571)
Supplement: S1 Supplementary Information — Non-dimensional forms of the full and static-cell models. (PDF) [file pone.0138571.s001.pdf]

S1 Supplementary Information. Mathematical model

S1.1 Non-dimensionalisation of full model

To reduce the number of parameters and systematically simplify the model the system of PDEs given by equations (2.1)-(2.8) was non-dimensionalised. Space, x, was rescaled with the length of the dorsal soft palette (DSP), L_P, while time, t, was rescaled with the maximum death rate of cells, Φ, leading to

$$x = L_P \hat{x},\ t = \frac{1}{\Phi}\hat{t}. \tag{S1.1}$$

This means that $\hat{x} \in [0,1]$ in DSP and $\hat{x} \in [0, \hat{L_T}]$ in the tongue (where $\hat{L_T} = L_T/L_P$) and $\hat{t} = 1$ represents approximately 3 hours. For the dependent variables

$$v = \beta L_P \hat{v},\ u = \beta L_P \hat{u},\ V_c = V_0 \hat{V_c},\ V_e = V_0 \hat{V_e},\ E = E_0 \hat{E},\ K = K_0 \hat{K}, \tag{S1.2}$$

which means that $\hat{E} \in [0,1]$ and $\hat{K} \in [0,1]$.

The dimensionless parameters are defined as follows

$$\begin{aligned}
&\hat{\beta} = \frac{\beta}{\Phi}, && \hat{\lambda} = \frac{\lambda}{\Phi}, && \hat{\delta} = \frac{\delta}{\Phi}, && \\
&\hat{\rho} = \frac{\rho V_0}{\Phi}, && \hat{\mu} = \frac{\mu}{\Phi}, && \hat{\gamma} = \frac{\gamma}{\Phi}, && \hat{\xi} = \frac{K_0 \xi}{V_0}, \\
&\hat{D}_V = \frac{D_V}{\Phi {L_P}^2}, && \hat{D}_E = \frac{D_E}{\Phi {L_P}^2}, && \hat{Q}_V = \frac{Q_V}{\Phi L_P}, && \hat{Q}_E = \frac{Q_E}{\Phi L_P} \\
&\hat{L}_T = \frac{L_T}{L_P}, && \hat{E}_B = \frac{E_B}{E_0}, && \hat{E}_G = \frac{E_G}{E_0}, && \hat{K}_{1/2} = \frac{K_{1/2}}{K_0},
\end{aligned} \tag{S1.3}$$

which leads to the following non-dimensional form of the full model,

$$S_e(\hat{x},\hat{t}) + S_c(\hat{x},\hat{t}) = 1, \tag{S1.4}$$

$$\frac{\partial S_c}{\partial \hat{t}} + \hat{\beta}\frac{\partial(\hat{v}S_c)}{\partial \hat{x}} = -\hat{f}(\hat{K})S_c + \hat{\beta}\hat{g_B}(\hat{E})S_c, \tag{S1.5}$$

$$\frac{\partial S_e}{\partial \hat{t}} + \hat{\beta}\frac{\partial(\hat{u}S_e)}{\partial \hat{x}} = \hat{f}(\hat{K})S_c - \hat{\beta}\hat{g_B}(\hat{E})S_c, \tag{S1.6}$$

$$\hat{D_E}\frac{\partial^2 \hat{E}}{\partial \hat{x}^2} = \hat{\lambda}S_c\hat{E} + \hat{\delta}\hat{E}, \tag{S1.7}$$

$$\frac{\partial(\hat{V}_cS_c)}{\partial \hat{t}} + \hat{\beta}\frac{\partial(\hat{v}\hat{V}_cS_c)}{\partial \hat{x}} = \hat{\xi}\hat{\rho}\hat{h_R}(\hat{E})\hat{g_G}(\hat{E})\hat{K}\hat{V}_cS_c + \hat{\mu}\hat{g_G}(\hat{E})\hat{h_U}(\hat{E})\hat{V}_eS_c - \hat{\gamma}\hat{V}_cS_c - \hat{f}(\hat{K})\hat{V}_cS_c, \tag{S1.8}$$

$$\frac{\partial(\hat{V}_eS_e)}{\partial \hat{t}} + \hat{\beta}\frac{\partial(\hat{u}\hat{V}_eS_e)}{\partial \hat{x}} = -\hat{\mu}\hat{g_G}(\hat{E})\hat{h_U}(\hat{E})\hat{V}_eS_c + \hat{\gamma}\hat{V}_cS_c + \hat{f}(\hat{K})\hat{V}_cS_c + \hat{D_V}\frac{\partial}{\partial \hat{x}}\left(S_e\frac{\partial \hat{V}_e}{\partial \hat{x}}\right), \tag{S1.9}$$

$$\frac{\partial(\hat{K}S_c)}{\partial \hat{t}} + \hat{\beta}\frac{\partial(\hat{v}\hat{K}S_c)}{\partial \hat{x}} = -\hat{K}\hat{h_R}(\hat{E})\hat{g_G}(\hat{E})\hat{V}_cS_c - \hat{K}\hat{f}(\hat{K})S_c + \hat{\beta}\hat{g_B}(\hat{K})S_c, \tag{S1.10}$$

where

$$\hat{g_B}(\hat{E}) = \frac{\hat{E}^{m_2}(1+\hat{E_B}^{m_2})}{\hat{E}^{m_2} + \hat{E_B}^{m_2}} \tag{S1.11}$$

$$\hat{g_G}(\hat{E}) = \frac{\hat{E}^{m_3}(1+\hat{E_G}^{m_3})}{\hat{E}^{m_3} + \hat{E_G}^{m_3}} \tag{S1.12}$$

$$\hat{h_R}(\hat{E}) = \rho_S + (\rho_B - \rho_S)\hat{g_B}(\hat{E}) \tag{S1.13}$$

$$\hat{h_U}(\hat{E}) = \mu_S + (\mu_B - \mu_S)\hat{g_B}(\hat{E}) \tag{S1.14}$$

$$\hat{f}(\hat{K}) = \frac{\hat{K}_{1/2}^{m_1}}{\hat{K}_{1/2}^{m_1} + \hat{K}^{m_1}}. \tag{S1.15}$$

S1.2 Non-dimensional static-cell model

The data in Table 2 suggest that $\hat{\beta} = \beta/\Phi \simeq 0.04$ (where β is the dimensional maximum rate of cell proliferation and Φ is the dimensional maximum rate of cell lysis due to viral infection), i.e. cell death due to viral infection occurs over much shorter time-scale than cell proliferation. Using $\hat{\beta} \ll 1$, and studying the leading order system as $\hat{\beta} \to 0$, yields:

$$S_e(x,t) + S_c(x,t) = 1, \tag{S1.16}$$

$$\frac{\partial S_c}{\partial t} = -f(K) S_c, \tag{S1.17}$$

$$\frac{\partial S_e}{\partial t} = f(K) S_c, \tag{S1.18}$$

$$D_E \frac{\partial^2 E}{\partial x^2} = \lambda S_c E + \delta E, \tag{S1.19}$$

$$\frac{\partial (V_c S_c)}{\partial t} = \xi \rho h_R(E) g_G(E) K V_c S_c + \mu g_G(E) h_U(E) V_e S_c - \gamma V_c S_c - f(K) V_c S_c, \tag{S1.20}$$

$$\frac{\partial (V_e S_e)}{\partial t} = -\mu g_G(E) h_U(E) V_e S_c + \gamma V_c S_c + f(K) V_c S_c + D_V \frac{\partial}{\partial x}\left(S_e \frac{\partial V_e}{\partial x}\right), \tag{S1.21}$$

$$\frac{\partial (K S_c)}{\partial t} = -K h_R(E) g_G(E) V_c S_c - K f(K) S_c, \tag{S1.22}$$

where hats have been dropped. This model is a reasonable approximation of the full model until $t = O(1/\beta) \simeq 75$ hours, which is beyond the timescale of interest $O(48)$ hours and by when the adaptive immune response becomes important. Consequently, the non-dimensional static-cell model has been used for the investigation of the FMDV infection dynamics.

Non-dimensional boundary and initial conditions. The initial conditions of the system in the non-dimensional static-cell model are

$$t = 0 : S_c = \alpha, S_e = 1 - \alpha, K = 1, V_c = 0, V_e(x,0) = V_0 \delta(x - e_p)$$

where δ is Dirac's delta function. On the basement membrane we have

$$x = 0 : E(0,t) = 1, -D_V \frac{\partial V_e}{\partial x}(0,t) = -Q_V V_e(0,t),$$

while the following apply on the DSP surface

$$x = L_P : -D_E \frac{\partial E}{\partial x}(L_P,t) = Q_E E(L_P,t),\ -D_V \frac{\partial V_e}{\partial x}(L_P,t) = Q_V V_e(L_P,t).$$

Boundary conditions for tongue on the granular-corneal layer boundary are

$$x = L_T : \frac{\partial E}{\partial x}(L_T,t) = 0,\ \frac{\partial V_e}{\partial x}(L_T,t) = 0.$$

The non-dimensional initial condition for activator concentration is:

$$E(x,0) = \frac{(A - \frac{Q_E}{D_E}) e^{-2A} e^{Ax}}{\frac{Q_E}{D_E}(1 - e^{2A}) + A(1 + e^{2A})} + \frac{(\frac{Q_E}{D_E} + A) e^{-Ax}}{\frac{Q_E}{D_E}(1 - e^{-2A}) + A(1 + e^{-2A})}, \tag{S1.23}$$

$0 \le x \le L_P$ in palate and

$$E(x,0) = \frac{e^{Ax}}{1 + e^{2AL_T}} + \frac{e^{2AL_T} e^{-Ax}}{1 + e^{2AL_T}}, \quad 0 \le x \le L_T \text{ in tongue}, \tag{S1.24}$$

where $A = \sqrt{(\lambda\alpha + \delta)/D_N}$.

References

[1] Kurosaka D, Nagamoto T. Inhibitory effect of TGF-β2 in human aqueous humor on bovine lens epithelial cell proliferation. Invest Ophthalmol Vis Sci. 1994; 35: 3408–3412.

[2] Masui H, Castro L, Mendelsohn J. Consumption of EGF by A431 cells: evidence for receptor recycling. J Cell Biol. 1993; 120: 85–93.

[3] Buckley A, Davidson JM, Kamerath CD, Woodward SC. Epidermal growth factor increases granulation tissue formation dose dependently. J Surg Res. 1987; 43: 322–328.

[4] Thorne RG, Hrabetova S, Nicholson C. Diffusion of epidermal growth factor in rat brain extracellular space measured by integrative optical imaging. J Neurophysiol. 2004; 92: 3471–3481.

[5] Monaghan P, Cook H, Jackson T, Ryan M, Wileman T. The ultrastructure of the developing replication site in foot-and-mouth disease virus-infected BHK-38 cells. J Gen Virol. 2004; 85: 933–946.

[6] Baranowski E, Sevilla N, Verdaguer N, Ruiz-Jarabo CM, Beck E, Domingo E. Multiple virulence determinants of foot-and-mouth disease virus in cell culture. J Virol. 1998; 72: 6362–6372.

[7] Breesex SSJ, Trautman R. Free diffusion measured by biological assay in multilayered cells: II. Diffusion coefficient of foot-and-mouth disease virus determined by infectivity. Anal Biochem. 1960; 1: 307–316.

[8] Schley D, Ward J, Zhang Z. Modelling foot-and-mouth disease virus dynamics in oral epithelium to help identify the determinants of lysis. Bull Math Biol. 2011; 73: 1503–1529. doi:10.1007/s11538-010-9576-6

Table S1.1. Non-dimensionalised model parameters, their interpretation and 'standard' values used in the simulations.

	Parameter	Value
$\hat{\beta}$	maximum rate of cell proliferation	3.99×10^{-2} [1]
$\hat{\lambda}$	uptake rate of activator by cells	5.92×10^{-12} [2]
$\hat{\delta}$	decay rate of activator	2.08 [3]
$\hat{D}_E$	diffusion coefficient of activator	19.1 [4]
$\hat{\xi}$	maximal replication rate of virus	2.65 (based on [5])
$\hat{\rho}$	rate at which virus uses up intracellular resource	4.14 (based on [5])
$\hat{\mu}$	virion-cell affinity and internalisation rate	6.01×10^{-5} (based on [6])
$\hat{\gamma}$	rate of virus release by live cells	0 (see S3 Supplementary Information)
$\hat{D}_V$	diffusion coefficient of virions	3.77 [7]
ρ_B	defines relative susceptibility of basal layer to FMDV replication	1 (see S3 Supplementary Information)
ρ_S	defines relative susceptibility of spinous layer to FMDV replication	1 (see S3 Supplementary Information)
μ_B	defines relative vulnerability of basal layer to FMDV infection	1 (see S3 Supplementary Information)
μ_S	defines relative vulnerability of spinous layer to FMDV infection	1 (see S3 Supplementary Information)
$\hat{K}_{1/2}$	value of K at which cell death is half maximum value	0.04 (see S3 Supplementary Information)
m_1	exponent in function $\hat{f}$ which defines resource depletion	4 (see S3 Supplementary Information)
m_2	exponent in function $\hat{g_B}$ which defines the basal layer	80 (see S3 Supplementary Information)
m_3	exponent in function $\hat{g_G}$ which defines the basal and spinous layers	80 (see S3 Supplementary Information)
$\hat{L}_P$	thickness of dorsal soft palate	1 (measured data)
$\hat{L}_T$	thickness of tongue	9.71 (measured data)
$\hat{L}_{Pb}$	threshold of spinous cell layer in dorsal soft palate	8.25×10^{-2} (measured data)
$\hat{L}_{Tb}$	threshold of spinous cell layer in tongue	7.13×10^{-2} (measured data)
$\hat{L}_{Tg}$	threshold of granular cell layer in tongue	9.3 (measured data)
$\hat{E}_B$	concentration of $\hat{E}$ at basal-spinous interface	9.66×10^{-1} (DSP) (based on measured data), 9.77×10^{-1} (tongue) (based on measured data)
$\hat{E}_G$	concentration of $\hat{E}$ at spinous-granular interface (DSP) or spinous-surface interface (tongue)	6.3×10^{-1} (DSP) (based on measured data), 8.18×10^{-2} (tongue) (based on measured data)

The values are either taken from the indicated source, measured as part of the study or estimated (see S3 Supplementary Information for details).

Table S1.2. Initial and boundary conditions parameters for the non-dimensionalised static-cell model.

	Parameter	Condition	Value
α	initial cellular space volume	$S_c(0,t)$	0.95 [8]
$\hat{E}_0$	activator at the basement membrane	$\hat{E}(0,t)$	1
$\hat{K}_0$	initial intracellular resource fraction per unit lenght	$\hat{K}(x,0)$	1 (see S3 Supplementary Information)
$\hat{V}_0$	viral infectious dose	$\hat{V}_e(e_p,0)$	1 (see S3 Supplementary Information)
$\hat{Q_E}$	activator mass transfer coefficient	$\frac{\partial \hat{E}}{\partial \hat{x}}(\hat{L_P})$	10 (see S3 Supplementary Information)
$\hat{Q_V}$	FMDV mass transfer coefficient	$\frac{\partial \hat{V}_e}{\partial \hat{x}}(\hat{L_P})$	5 (see S3 Supplementary Information)
e_p	viral entry point	various points tested	0, 0.175, $\hat{L}_i$ or $\hat{L}_i - 0.175$, where i=P,T

See S3 Supplementary Information for details.
