# Supplementary material for: Using Mathematical Modelling to Explore Hypotheses about the Role of Bovine Epithelium Structure in Foot-And-Mouth Disease Virus-Induced Cell Lysis
Source: PLoS One. 2015 Oct 2;10(10):e0138571. doi: 10.1371/journal.pone.0138571 (PMC4592007; doi:10.1371/journal.pone.0138571)
Supplement: S2 Supplementary Information — Details on the bovine epithelial cell data collection and analysis. (PDF) [file pone.0138571.s002.pdf]

S2 Supplementary Information.
Epithelial thickness and cell layer structure

The absence of information about the tongue and dorsal soft palette (DSP) epithelial tissues motivated the collection of epithelial cell data relating to the thickness and cell layer structure of these tissues in order to estimate relevant parameters of the model. Data on cell density and dimensions in each cell layer were also collected. The raw data are provided in S1 Data Supplement.

S2.1 Tissue samples and data collection

Two uninfected, H&E (haematoxylin and eosin) stained bovine tissue slides were used; one of cattle soft palate and one of cattle tongue tissue (The Pirbright Institute slides 2002/3190 and 2003/2001 respectively). Measurements were made using a Leica AS LMD microscope and Leica Laser Microdissection V 4.4 software. Data on cell height and width, epithelial tissue thickness and number of adjacent cells were collected manually utilising the features of the microscope software.

Considering the continuous nature of epithelium and the unclear distinction between different cell layers, in combination with the vast area covered by the spinous layer, a distinction between different spinous sub-layers was made to aid the collection of data. These are the lower, middle and upper spinous sub-layers. Due to the limited number of the examined tissue slides, a manual approach in the collection of data was considered to be the most efficient. For a higher number of samples it would be worthwhile to select relevant imaging processing software. Figure S2.1(a) shows a sample image of cell measurements of the middle spinous layer of the tongue. Sample images of DSP tissue adjacent cell estimates and tissue thickness are given in Figures S2.1(b) and S2.1(c) respectively.

S2.2 Cell size measurements

Measurements of height and width of adjacent cells in each layer/sub-layer were taken. Ten representative areas were selected for each layer/ sub-layer and measurements of 10 adjacent cells were recorded. In total, data on at least 100 cells from each layer or sub-layer were collected. Cell width is considered to be the measurement taken in parallel to the epithelium and cell height the measurement which is perpendicular to width. However, due to the nature of epithelial cells and the structure of epithelium, the identification of height and width was not always straightforward. Furthermore, some samples taken from the upper spinous layer of DSP contained fewer than 10 cells, therefore an increased number of samples (15) was needed in order to measure at least 100 cells in total. The sample taking procedure was also adjusted for samples taken from the lower spinous and middle spinous sub-layers of tongue. This is due to the thickness of these tongue sub-layers, in combination with the magnification needed to identify cells and cells' borders, impeding taking images of the whole sample. As a result, a number of adjacent sub-samples were taken which, when put together, depict the whole thickness of each sub-layer. Measured cells in these sub-samples were selected to be adjacent in order to represent the whole sub-layer. Due to the high number of cells contained in these samples, only five representative regions were selected for cell measurements in the tongue lower

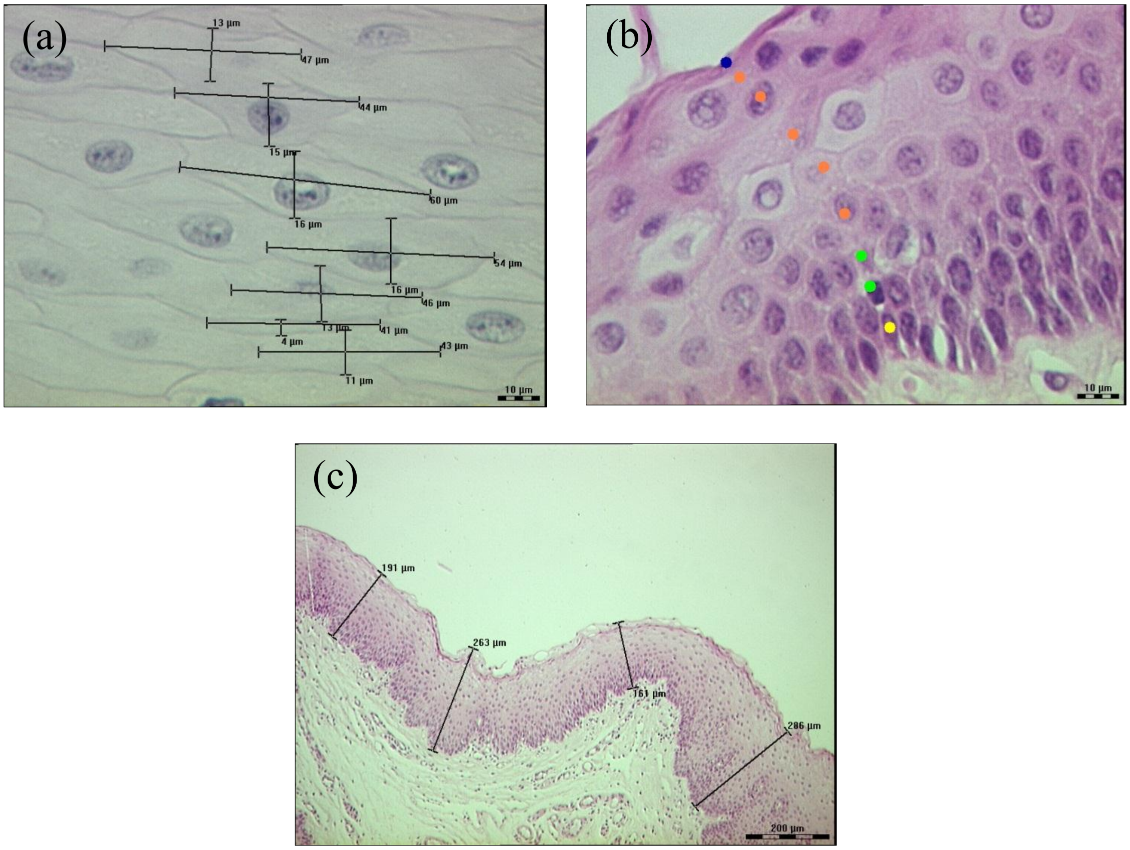

Fig. S2.1. Example images of epithelial thickness and cell layer structure measurements of uninfected cattle tongue epithelium. (a) Measurements of height and width of selected middle spinous cells (slide 2003/2001). (b) Estimation of number of adjacent cells in each epithelial tissue layer. Yellow dots illustrate the counted basal cells, green dots the lower spinous cells, orange dots the middle spinous cells and blue dots the upper spinous cells (slide 2002/3190). (c) Measurements of tissue thickness (slide 2002/3190).

and middle spinous sub-layers. Even then, the number of measured cells still exceeded 100 in both cases (386 in lower spinous and 164 in middle spinous). Height and width were measured in μm on a scale of 1cm:10μm magnification.

S2.3 Adjacent cells measurements

Ten representative areas, distinct from those used before, were selected and the total number of adjacent cells spanning the whole depth of the tissue was estimated. The layer/sub-layer of each cell was also recorded. Scale of magnification used for all samples was 2.25cm:500μm.

S2.4 Epithelium thickness measurements

Twenty sites along the width of each tissue were selected to measure epithelium thickness. The selection of these sites was unrelated to the areas selected for cell size or adjacent

cell measurements. In the DSP sites were chosen so as to be spread throughout the width of the tissue and to ignore areas where the tissue was damaged during processing. For the tongue samples these points were more accurately spaced throughout the tissue to be equidistant from each other. In addition to the whole epithelium thickness measurements, thickness of the tongue's basal-spinous epithelial tissue were recorded. Measurements were taken in μm. Scale of magnification used for DSP was 2.25cm:200μm and for tongue was 2.25cm:500μm.

S2.5 Results

Cell height and width data were used to estimate the average cell height and width (10.5×10^{-4} cm and 25.6×10^{-4} cm respectively). These were used in the estimation of parameters of the model (see S3 Supplementary Information). Cell height and width data are summarised in Figure S2.2. For the estimation of the epithelial tissue thickness of tongue and DSP individual cell layer thickness was summed to basal-spinous-granular thickness (see Table S2.1). These estimates are in good agreement with the direct measurements of epithelium thickness (see section S2.4 and S1 Data Supplement). Although different estimates of mean cell height (in each cell layer) have been used in the estimation of DSP and tongue thickness (see Table S2.1), no other differences between DSP and tongue have been included in the model so as to maintain simplicity.

Table S2.1. Collected data of individual epithelial cell layer thickness from uninfected, H&E (haematoxylin and eosin) stained bovine tissue slides 2003/2001 (tongue) and 2002/3190 (DSP), measured in microns and rounded to three significant figures.

	Epithelial cell layer thickness (μm)					Epithelium thickness
Epithelial tissue type	Basal	Lower spinous	Middle spinous	Upper spinous	Granular	
DSP	14.1	59.8	78.5	18.9	N/A	171
Tongue	12.2	1080	419	82.9	65.1	1659

Data were compiled using the mean number of cells in each cell layer and the mean height of each cell in that particular layer. Epithelium thickness is estimated by adding the individual cell layer thickness (excludes corneal layer).

Cell width measurements are similar in both DSP and tongue for all layers. Cell height is generally smaller for DSP cells, though interlayer differences are also evident with basal cells in both tissues to have considerably lower height measurements. In DSP, lower spinous cells have similar height as basal cells.

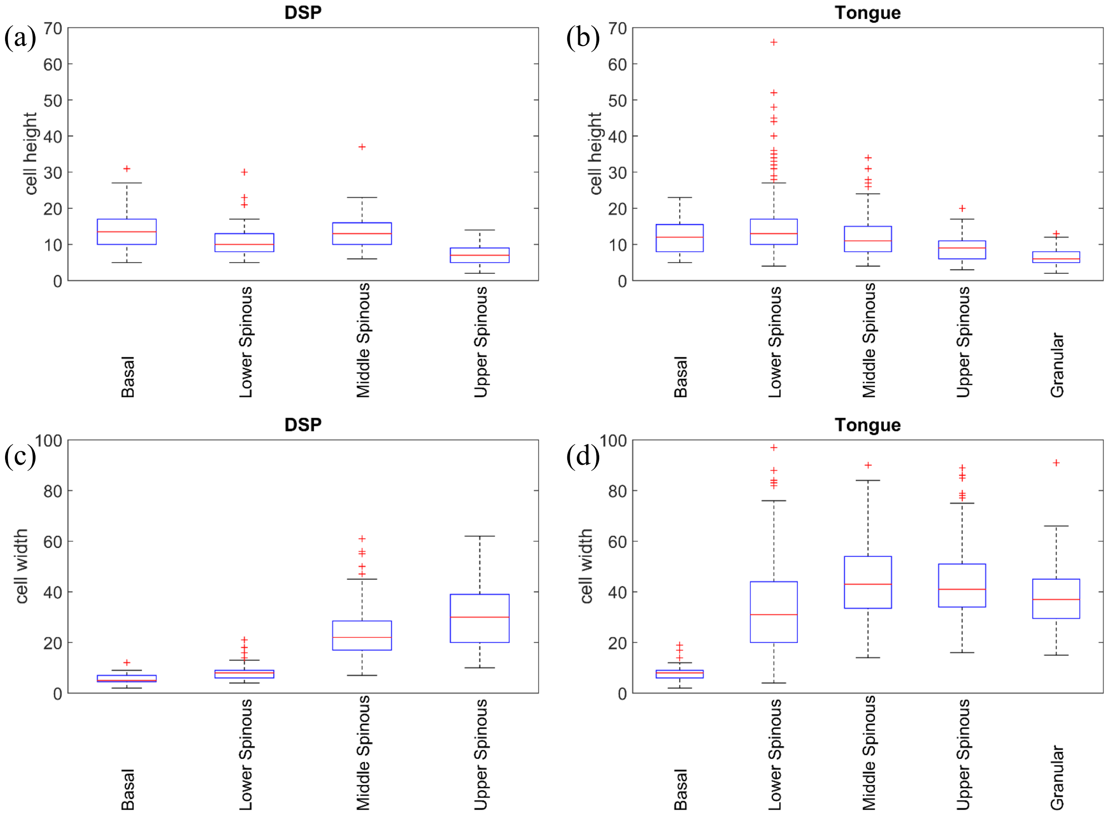

Fig. S2.2. Statistical analysis of epithelial cell data. Epithelial cell measurements data of uninfected dorsal soft palate (DSP) and tongue tissues, classified into basal, lower spinous, middle spinous, upper spinous and granular layers. Central mark (red line) indicates the median value, box edges the 25th and 75th percentiles and whiskers provide a 99.3 data coverage. Outliers are marked in red. (a), (b) Cell height measurements (μm) in the DSP and tongue respectively. An outlier of 159μm (and its impact) was excluded from the displayed data of tongue lower spinus cell height. (c), (d) Cell width measurements (μm) in the DSP and tongue respectively.
