# Supplementary material for: Using Mathematical Modelling to Explore Hypotheses about the Role of Bovine Epithelium Structure in Foot-And-Mouth Disease Virus-Induced Cell Lysis
Source: PLoS One. 2015 Oct 2;10(10):e0138571. doi: 10.1371/journal.pone.0138571 (PMC4592007; doi:10.1371/journal.pone.0138571)
Supplement: S3 Supplementary Information — Detailed explanation of the parameter estimates derivation. (PDF) [file pone.0138571.s003.pdf]

S3 Supplementary Information. Parameter estimation

The majority of the parameters were estimated using data from the literature. However, no relevant data on epithelial tissue structure existed, so this was collected to inform some of the parameters (see S2 Supplementary Information). The parameters of tissue thickness, L_P, L_T, and cell layer structure, L_{Pb}, L_{Tb}, L_{Tg}, E_B and E_G, were estimated based on this data. Also the intracellular resource parameter estimation for K_0 and $K_{1/2}$ was based on this data, while the activator parameter λ was estimated using both literature and data collected as part of this study. A reduced model in combination with collected and literature data was used for the viral replication parameters ξ and ρ. Parameter estimates described in this section are the baseline estimates, but sensitivity of the model output to parameter values was explored (see section 3).

S3.1 Initial and boundary conditions

α - initial space fraction of cellular space

The cellular space fraction of a healthy epithelial tissue was estimated by Schley *et al.* to be 0.95 [1], based upon data presented by Moore [2].

E_0 - amount of activator at the basement membrane

The activator amount at the basement membrane (entry point) was set to be 1 arbitrary unit per cm.

K_0 - initial intracellular resource fraction per unit length

Intracellular resource, K, is quantified as resource per cell column height, meaning the fraction of surviving cell resource per cell column height. The average height of epithelial cells as derived by the average height of tongue (12.5μm) and DSP epithelial cells (8.41μm), is 10.5μm (see section 2.4.1). In the absence of virus intracellular resource is intact, thus the fraction of surviving resource is 1 and consequently its concentration is given by,

$$K_0 = \frac{1}{10.5 \times 10^{-4}\ \text{cm}} = 952\ \text{cm}^{-1}.$$

V_0 - quantity of FMDV entering the system

The amount of virus entering the system at time point zero was defined as 1 PFU in total, which equates to a concentration of 2920 PFU per cm of cell height in our model if viral entry occurs over one non-dimensional spatial step (here 3.42×10^{-4} cm in dimensional form). For an average cell height of 10.5×10^{-4} cm this corresponds to an initial concentration of approximately 3.07 PFU per cell.

e_p - FMDV entry point

Four different viral entry points were used in the simulations: (i) the basement membrane; (ii) the tissue surface (DSP) or the boundary between granular and corneal layers (tongue); (iii) about two cells deep from the basement membrane; and (iv) about two cells deep from the tissue surface or the granular-corneal boundary. Parameter e_p takes the values 0, L_i, 3×10^{-3}cm and $L_i - 3 \times 10^{-3}$ cm respectively, where i=P, T (see S3.2 and S3.2 for tongue and DSP epithelial thickness).

The basement membrane was chosen as an entry point because tongue is usually infected through viraemia [3]. It has been suggested though that virus may be delivered to a point in the spinous layer, which could act as the first site of infection [4]. The DSP is a site of primary infection [3], therefore the tissue surface in this case or the boundary between granular and corneal layers in the case of tongue was selected as a possible site of infection. A cut or abrasion may result to a viral entry point a few cells deep from the DSP surface. As the corneal layer of the tongue is assumed to be impenetrable by FMDV, a cut will have to reach the granular or spinous layer in order to result to infection. Such cuts or abrasions have been reported in the literature to be a route of infection [3].

Q_E - mass transfer coefficient of activator E

In the absence of data, Q_E was estimated based on the equilibrium equation of E for DSP (see (2.14)) and the derived estimations of E_B and E_G. In non-dimensional equations we have

$$\begin{aligned} E_B &= E(L_{Pb}, 0) &= \frac{(A - \frac{Q_E}{D_E})e^{-2A}e^{AL_{Pb}}}{\frac{Q_E}{D_E}(1 - e^{2A}) + A(1 + e^{2A})} + \frac{(\frac{Q_E}{D_E} + A)e^{-AL_{Pb}}}{\frac{Q_E}{D_E}(1 - e^{-2A}) + A(1 + e^{-2A})} \\ E_G &= E(L_P, 0) &= \frac{(A - \frac{Q_E}{D_E})e^{-2A}e^{AL_P}}{\frac{Q_E}{D_E}(1 - e^{2A}) + A(1 + e^{2A})} + \frac{(\frac{Q_E}{D_E} + A)e^{-AL_P}}{\frac{Q_E}{D_E}(1 - e^{-2A}) + A(1 + e^{-2A})} \end{aligned}$$

where $A = \sqrt{(\lambda\alpha + \delta)/D_E}$. As parameters E_B and E_G depend on Q_E and they must differ sufficiently to separate the basal and spinous layers of the tissue, an appropriate value for Q_E was selected. The non-dimensional value of Q_E is 10.

Q_V - mass transfer coefficient of FMDV

In the absence of relevant data and as activator diffusion is much higher than virus diffusion, it was assumed that the mass transfer coefficient of FMDV is also lower than the relevant activator parameter. The non-dimensional value of Q_V was set to 5 but a variety of other values were explored in the sensitivity analysis of the model.

S3.2 Model parameters

β - maximum rate of bovine epithelial cell proliferation

The rate of cell proliferation has been estimated as 1.33×10^{-2} h^{-1} for lens epithelial cells. The initial number of cells was 6700 in each well, which after 96 hours of proliferation yielded 24000 cells [5]. Assuming

$$\frac{dN}{dt} = \beta N \Rightarrow N = N_0 \exp \beta t.$$

Since $N_0 = 6700$ and $N(96) = 24000$, then

$$\beta = \frac{\ln(\frac{N(96)}{N_0})}{96} \simeq 0.0133 \text{ h}^{-1}.$$

The rate of cell proliferation is considered to be negligible over the timescale of interest, since $\hat{\beta} \ll 1$ (see section S1.2).

λ - uptake rate of activator by cells

Data on epithelial growth factor (EGF) were used to estimate the activator parameters. Masui *et al.* (1993) estimated consumption of radio-labelled EGF by A431 cells, a human squamous carcinoma cell line, testing various concentrations [6]. Based on these data the EGF uptake rate was estimated to be within the range of $[3.67 \times 10^{-19}, 2.92 \times 10^{-18}]$ mol/h/cell, depending on EGF concentration used. For parameter λ the maximum estimate was used, which responds to an EGF concentration of 215 nM. The volume of an epithelial cell is (height$\times$width^2) which based on the average cell height and width of bovine epithelial cells is equal to 6.88×10^{-9} cm^3 (see S2 Supplementary Information). Hence, the uptake rate is,

$$\lambda = 2.92 \times 10^{-18} \frac{\text{mol}}{\text{cell} \times \text{h}} \times \frac{1}{215} \frac{\text{cm}^3}{\text{mol}} \times \frac{1}{6.88 \times 10^{-9}} \frac{\text{cell}}{\text{cm}^3} = 1.97 \times 10^{-12} \text{ h}^{-1}$$

δ - decay rate of activator

The estimated EGF half life is about one hour [7]. Hence

$$\frac{\ln 2}{1} = 0.693 \text{ h}^{-1}$$

D_E - diffusion coefficient of activator

For the diffusion coefficient of the activator, data of the EGF diffusion in rat somatosensory cortex was used [8]. The diffusion coefficient for EGF was estimated to be to be $5.18 \pm 0.16 \times 10^{-7}$ cm^2/s [8] and, hence, $D_E = 1.86 \times 10^{-3}$ cm^2/h.

Φ - maximum rate of cell lysis due to viral infection

The results of Monaghan *et al.* (2003) [9] indicate that widespread cell death can be seen as early as 3 hours post infection with FMDV, which is equal to a rate of 3.33×10^{-1} h^{-1}.

$K_{1/2}$ - threshold parameter for cell death

This is the threshold concentration of resource at which a cell moves from a healthy to a dying state. In the absence of relevant data its estimate was set based on experimental observations of the authors. It is expected that cells die when most of their resource is consumed, thus $K_{1/2}$ was set at $0.04 \times K_0 = 38.1$ cm^{-1}.

ξ - maximal replication rate of FMDV

The maximal replication rate of FMDV was estimated using a reduced model which assumes the cellular space fraction to be 1 (see S4 Supplementary Information). The reduced model equations give

$$V_c(t_1) + \xi K(t_1) = V_c(t_2) + \xi K(t_2),$$

which leads to an estimate for ξ based on observed concentrations of intracellular virus and intracellular resource at two different time points. Data show viral concentration for FMDV O serotype at one hour post infection to be $V_c(1) = 4 \times 10^4$ PFU/ml and at three hours post infection to be $V_c(3) = 6 \times 10^7$ PFU/ml [10]. In the absence of information on the number of cells used in the experiments of the study providing this data, an approximation of cells per ml was employed assuming that volume equates to cell volume (see section S3.2 for the estimation of cell volume). The average cell height was then used to modify the units to PFU per cm (see section 2.4.1). Therefore

$$V_c(1) = 4 \times 10^4 \frac{\text{PFU}}{\text{ml}} \times \frac{1}{4 \times 10^8} \frac{\text{ml}}{\text{cell}} \times \frac{1}{10.5 \times 10^{-4}} \frac{\text{cell}}{\text{cm}} = 9.52 \times 10^{-2} \frac{\text{PFU}}{\text{cm}},$$

$$V_c(3) = 6 \times 10^7 \frac{\text{PFU}}{\text{ml}} \times \frac{1}{4 \times 10^8} \frac{\text{ml}}{\text{cell}} \times \frac{1}{10.5 \times 10^{-4}} \frac{\text{cell}}{\text{cm}} = 1.43 \times 10^2 \frac{\text{PFU}}{\text{cm}}.$$

Monaghan *et al.* (2004) mention that extensive cytopathic effect (CPE) and thus extensive cell death occurs at about three hours post infection [10]. Using this information it is assumed that at three hours post infection intracellular resource, K, reaches its threshold value $K_{1/2}$ which signifies the turning point for the initiation of high level cell death. Assuming also that intracellular resource at one hour post infection is nearly intact ($K(1) = K_0$), the parameter ξ was estimated to be equal to 1.56×10^{-1} PFU/resource.

ρ - rate at which FMDV uses up intracellular resource

The reduced model employed for the estimation of ξ (see S4 Supplementary Information), was also used for the estimation of ρ. In this case,

$$\rho = \frac{a}{\xi K}$$

was used to estimate ρ, where

$$a = \frac{1}{t_2 - t_1} ln \frac{V_c(t_2)}{V_c(t_1)}.$$

The same data on intracellular virus and resource used to estimate ξ, were also employed here [10] . The quantity a was estimated to be equal to 3.66 h^{-1}, leading to an estimate of $\rho = 2.46 \times 10^{-2}$ $((\text{PFU/cm}) \times \text{h})^{-1}$.

μ - virion-cell affinity and internalisation rate

Data for estimating this parameter where obtained from a study where a BHK-21 cell culture was infected with FMDV serotype C [11]. In this experiment 2×10^6 cells were infected with multiplicity of infection (MOI) of 5 PFU per cell. There was then an absorption period of 1 hour at 37°C, after which the viral load was 2×10^2 PFU. Using this data it was estimated that

$$\mu = \frac{\text{absorbed PFU}}{\text{inoculated PFU} \times \text{hours}} = \frac{2 \times 10^2 \text{ PFU}}{5 \times 2 \times 10^6 \text{ PFU} \times \text{h}} = 2 \times 10^{-5}\text{h}^{-1}.$$

γ - rate of non-lytic FMDV release

Release of FMDV by live cells was reported to occur to some extent in neutrophils from calf tonsil epithelia [12], but since neutrophils are white blood cells this information is not considered relevant to our model. A further report of non-lytic FMDV release is found in Blackwell *et al.* [13] who reported that bovine mammary gland secretory cells released FMDV while alive. The authors suggest this happens through exocytotic cell mechanisms which are normally used for the production and release of milk products. Given the specialised nature of these mechanisms, this information is also not considered relevant to this work. The absence of appropriate data relevant to epithelial cells for such a well-studied disease [14, 15] is considered indicative of the absence of live cell release. With this in mind, the rate of non-lytic FMDV release was set to be equal to zero but other values were also explored.

D_V - diffusion coefficient of FMDV

The diffusion coefficient for FMDV was estimated to be $3.67 \times 10^{-4}\text{cm}^2/\text{h}$ [16].

ρ_B, ρ_S - define the vulnerability of the basal and spinous cell layers respectively to FMDV replication

As both basal and spinous layers are vulnerable to FMDV infection and in the absence of information about differences in viral replication between these, we assume they have the same level of vulnerability to FMDV replication. Parameters ρ_B and ρ_S were therefore both assigned the value 1, though differences between layers have been explored in the sensitivity analysis of the model.

μ_B, μ_S - define the vulnerability of the basal and spinous cell layers respectively to FMDV infection

Monaghan *et al.* (2005) presented data suggesting differences in the expression of integrin αvβ6 between basal and spinous cells [17]. The authors have also proposed αvβ6 is the major receptor determining tropism in FMDV infected epithelial tissues, based on data on its presence in tissues such as the tongue and its absence from DSP. Nevertheless, for the purpose of this work, and because other receptors have also been shown to facilitate FMDV infection of epithelia, tongue and DSP are assumed to have the same virion-cell affinity and internalisation rate, μ. Furthermore, the differences in the expression of αvβ6 between basal and spinous cells has not been quantified, so both layers are assumed to

have the same vulnerability to infection. Parameters μ_B and μ_S are both assigned the value 1 as a starting point for our investigation, though differences between layers are explored as part of the sensitivity analysis (see section 3.4).

m_1 **- exponent in function** f

This parameter, which defines the rate of transition of cell death, was set to be 4 given the assumption that the transition from the healthy cell to the dying state is relatively sharp.

m_2**,** m_3 **- exponents in functions** g_B **and** g_G **respectively**

Both parameters were assigned the value 80, as it is assumed functions g_B and g_G change values rapidly at the threshold between basal and spinous layers, and spinous and granular layers respectively. The value assigned to the parameters was the minimum required in order to have both $g_B(E)$ and $g_G(E) \in [0, 1]$, for $E \in [0, E_0]$.

L_P **- thickness of dorsal soft palate**

The average thickness of dorsal soft palate was estimated from data on the average number of cells per layer and the average height of cells in each layer to be 1.71×10^{-2}cm (see Table S2.1).

L_T **- thickness of tongue**

The average thickness of basal-spinous-granular tongue epithelial tissue was estimated from data on the average number of cells per layer and the average height of cells in each layer to be 1.66×10^{-1}cm (see Table S2.1). The corneal layer was excluded from this estimate because individual cells cannot be identified.

L_{Pb} **- threshold of spinous cell layer in dorsal soft palate**

This is the average thickness of the basal layer in the dorsal soft palate. It was estimated using data on the average number of cells in the basal layer and the average height of cells in this layer and is 1.41×10^{-3}cm (see Table S2.1).

L_{Tb} **- threshold of spinous cell layer in tongue**

This is the average thickness of the basal layer in the dorsal soft palate. It was estimated using data on the average number of cells in the basal layer and the average height of cells in this layer and is 1.22×10^{-3}cm (see Table S2.1).

L_{Tg} **- threshold of granular cell layer in tongue**

This is the average thickness of the basal and spinous layers in tongue combined. It was estimated using data on the average number of cells per layer and the average height of cells in each layer and is 1.59×10^{-1}cm (see Table S2.1).

References

[1] Schley D, Ward J, Zhang Z. Modelling foot-and-mouth disease virus dynamics in oral epithelium to help identify the determinants of lysis. Bull Math Biol. 2011; 73: 1503–1529. doi:10.1007/s11538-010-9576-6

[2] Moore JV. Death of cells and necrosis in tumours. In: Potten CS editor. Perspectives on mammalian cell death. Oxford: Oxford University Press; 1987. pp. 295-325

[3] Alexandersen S, Zhang Z, Donaldson AI, Garland AJM. The pathogenesis and diagnosis of foot-and-mouth disease. J Comp Pathol. 2003; 129: 1–36.

[4] Monaghan P, Simpson J, Murphy C, Durand S, Quan M, Alexandersen S. Use of confocal immunofluorescence microscopy to localize viral nonstructural proteins and potential sites of replication in pigs experimentally infected with foot-and-mouth disease virus. J Virol. 2005; 79: 6410–6418.

[5] Kurosaka D, Nagamoto T. Inhibitory effect of TGF-β2 in human aqueous humor on bovine lens epithelial cell proliferation. Invest Ophthalmol Vis Sci. 1994; 35: 3408–3412.

[6] Masui H, Castro L, Mendelsohn J. Consumption of EGF by A431 cells: evidence for receptor recycling. J Cell Biol. 1993; 120: 85–93.

[7] Buckley A, Davidson JM, Kamerath CD, Woodward SC. Epidermal growth factor increases granulation tissue formation dose dependently. J Surg Res. 1987; 43: 322–328.

[8] Thorne RG, Hrabetova S, Nicholson C. Diffusion of epidermal growth factor in rat brain extracellular space measured by integrative optical imaging. J Neurophysiol. 2004; 92: 3471–3481.

[9] Monaghan P, Cook H, Hawes P, Simpson J, Tomley F. High-pressure freezing in the study of animal pathogens. J Microsc. 2003; 212: 62–70.

[10] Monaghan P, Cook H, Jackson T, Ryan M, Wileman T. The ultrastructure of the developing replication site in foot-and-mouth disease virus-infected BHK-38 cells. J Gen Virol. 2004; 85: 933–946.

[11] Baranowski E, Sevilla N, Verdaguer N, Ruiz-Jarabo CM, Beck E, Domingo E. Multiple virulence determinants of foot-and-mouth disease virus in cell culture. J Virol. 1998; 72: 6362–6372.

[12] Brown CC, Meyer RF, Olander HJ, House C, Mebus C. A pathogenesis study of foot-and-mouth disease in cattle, using in situ hybridization. Can J Vet Res. 1992; 56: 189–193

[13] Blackwell J, Wool S, Kosikowski F. Vesicular exocytosis of foot-and-mouth disease virus from mammary gland secretory epithelium of infected cows. J Gen Virol. 1981; 56: 207–212.

[14] Arzt J, Juleff N, Zhang Z, Rodriguez LL. The pathogenesis of foot-and-mouth disease I: viral pathways in cattle. Transbound Emerg Dis. 2011; 58: 291–304. doi:10.1111/j.1865-1682.2011.01204.x

[15] Grubman MJ, Baxt B. Foot-and-mouth disease. Clin Microbiol Rev. 2004; 17: 465–493.

[16] Breesex SSJ, Trautman R. Free diffusion measured by biological assay in multilayered cells: II. Diffusion coefficient of foot-and-mouth disease virus determined by infectivity. Anal Biochem. 1960; 1: 307–316.

[17] Monaghan P, Gold S, Simpson J, Zhang Z, Weinreb PH, Violette SM, et al. The αvβ6 integrin receptor for foot-and-mouth disease virus is expressed constitutively on the epithelial cells targeted in cattle. J Gen Virol. 2005; 86: 2769–2780.
