# Supplementary material for: Using Mathematical Modelling to Explore Hypotheses about the Role of Bovine Epithelium Structure in Foot-And-Mouth Disease Virus-Induced Cell Lysis
Source: PLoS One. 2015 Oct 2;10(10):e0138571. doi: 10.1371/journal.pone.0138571 (PMC4592007; doi:10.1371/journal.pone.0138571)
Supplement: S4 Supplementary Information — (PDF) [file pone.0138571.s004.pdf]

S4 Supplementary Information.
Reduced model for the estimation of ρ and ξ.

In the light of relevant *in vitro* experimental FMDV data [1], the maximal replication rate of FMDV, ξ, and the rate at which the virus consumes the intracellular resource, ρ, were estimated using a simplified, non-spatial form of the dimensional model. This reduced system is used only for the purpose of estimating ξ and ρ and it does not have a biologically realistic structure.

In this system we only account for cellular space ($S_c = 1$) and assume that no transfer of FMDV occurs between spaces (no viral uptake by cells, no live cell release, so $\mu = \gamma = 0$). Furthermore, we consider that the cellular space belongs to the basal-spinous epithelium ($g_G = 1$) and viral replication occurs at the same rate for any point of the cellular space ($h_R = 1$). The equations of the simplified system are then,

$$\frac{\partial S_c}{\partial t} = -\Phi f(K) S_c, \tag{S4.1}$$

$$\frac{\partial (V_c S_c)}{\partial t} = \xi \rho K V_c S_c - \Phi f(K) V_c S_c, \tag{S4.2}$$

$$\frac{\partial (K S_c)}{\partial t} = -\rho K V_c S_c - \Phi K f(K) S_c. \tag{S4.3}$$

Combining equations (S4.1) - (S4.3) we can reduce the system even further to

$$\frac{\partial V_c}{\partial t} = \xi \rho K V_c, \text{ and} \tag{S4.4}$$

$$\frac{\partial K}{\partial t} = -\rho K V_c, \tag{S4.5}$$

which together lead to

$$\frac{\partial (V_c + \xi K)}{\partial t} = 0. \tag{S4.6}$$

This means that

$$V_c(t_1) + \xi K(t_1) = V_c(t_2) + \xi K(t_2), \tag{S4.7}$$

can be used to estimate parameter ξ, given known values of cellular virus and intracellular resource (see paragraphs on estimation of ξ and ρ in section S3.2).

To estimate ρ it is assumed that there is exponential growth of virus. This assumption is relevant to the initial stages of infection, when resource is abundant and it can be assumed that the per capita rate of virus replication is constant. We have that,

$$\frac{\partial V_c}{\partial t} = a V_c \tag{S4.8}$$

and

$$V_c(t_2) = V_c(t_1) e^{(a(t_2 - t_1))}. \tag{S4.9}$$

Rearranging equation (S4.9) gives

$$a = \frac{1}{t_2 - t_1} \ln \frac{V_c(t_2)}{V_c(t_1)}. \tag{S4.10}$$

Applying data of viral growth [1, 2] to (S4.10), and combining (S4.4) and (S4.8) we obtain an estimate for ρ:

$$\rho = \frac{a}{\xi K}. \tag{S4.11}$$

Equations (S4.7) and (S4.11) are therefore used in section S3.2 to obtain estimates for parameters ρ and ξ.

References

[1] Monaghan P, Cook H, Jackson T, Ryan M, Wileman T. The ultrastructure of the developing replication site in foot-and-mouth disease virus-infected BHK-38 cells. J Gen Virol. 2004; 85: 933–946.

[2] Baranowski E, Sevilla N, Verdaguer N, Ruiz-Jarabo CM, Beck E, Domingo E. Multiple virulence determinants of foot-and-mouth disease virus in cell culture. J Virol. 1998; 72: 6362–6372.
