# Supplementary material for: Using Mathematical Modelling to Explore Hypotheses about the Role of Bovine Epithelium Structure in Foot-And-Mouth Disease Virus-Induced Cell Lysis
Source: PLoS One. 2015 Oct 2;10(10):e0138571. doi: 10.1371/journal.pone.0138571 (PMC4592007; doi:10.1371/journal.pone.0138571)
Supplement: S5 Supplementary Information — (PDF) [file pone.0138571.s005.pdf]

S5 Supplementary Information.

Supplementary results.

Here we provide additional simulation results discussed in the main paper.

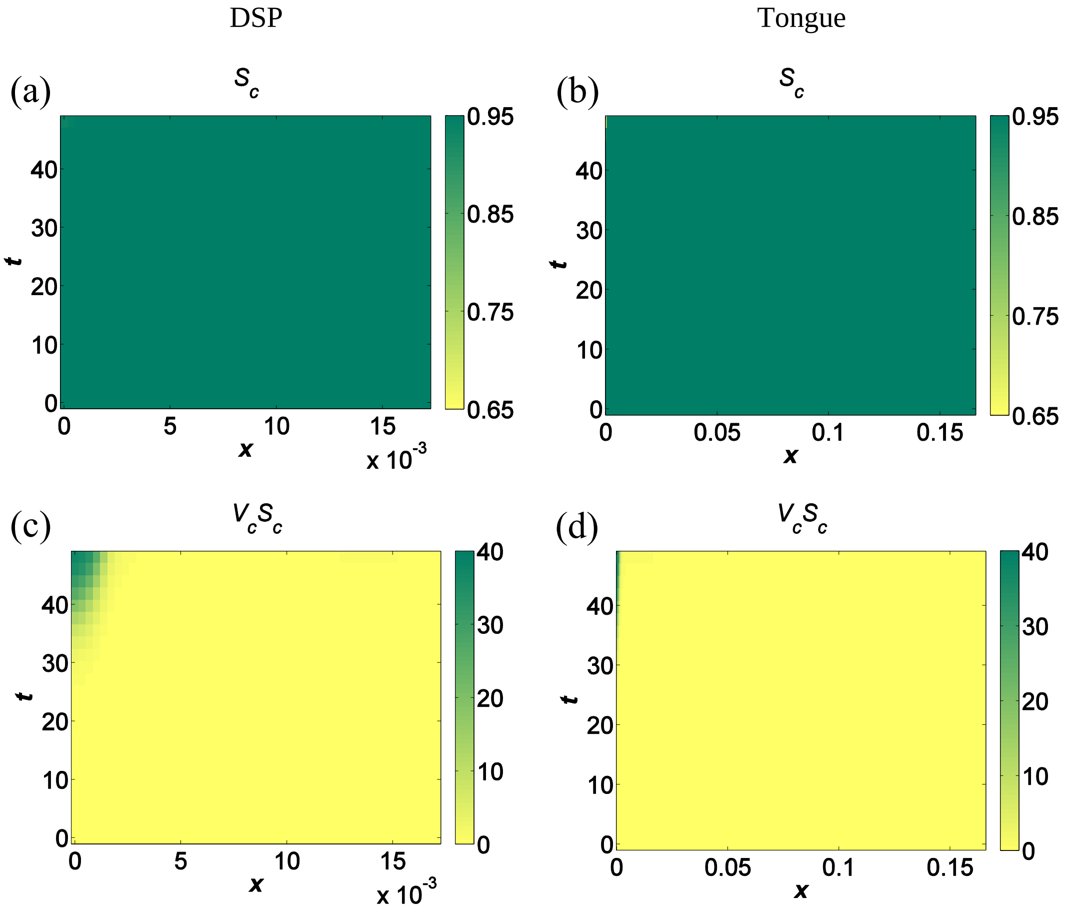

Fig. S5.1. Simulation results for altered viral replication parameter estimates. Results for DSP and tongue over a 48 hour timescale for maximal replication rate, ξ, and rate of FMDV resource consumption, ρ, both set at $0.29\times$ their default estimates. Viral replication in the spinous layer is defined here as half that of the basal layer. Epithelium surface used as the viral entry point for DSP and basement membrane as the viral entry point for tongue. (a), (b) Cellular space fraction, S_c, of DSP and tongue respectively. Reduced levels of cellular space fraction are observed at the top left hand corner of graph (b). (c), (d) Intracellular virus load, V_cS_c, of DSP and tongue respectively measured in PFU/cm.

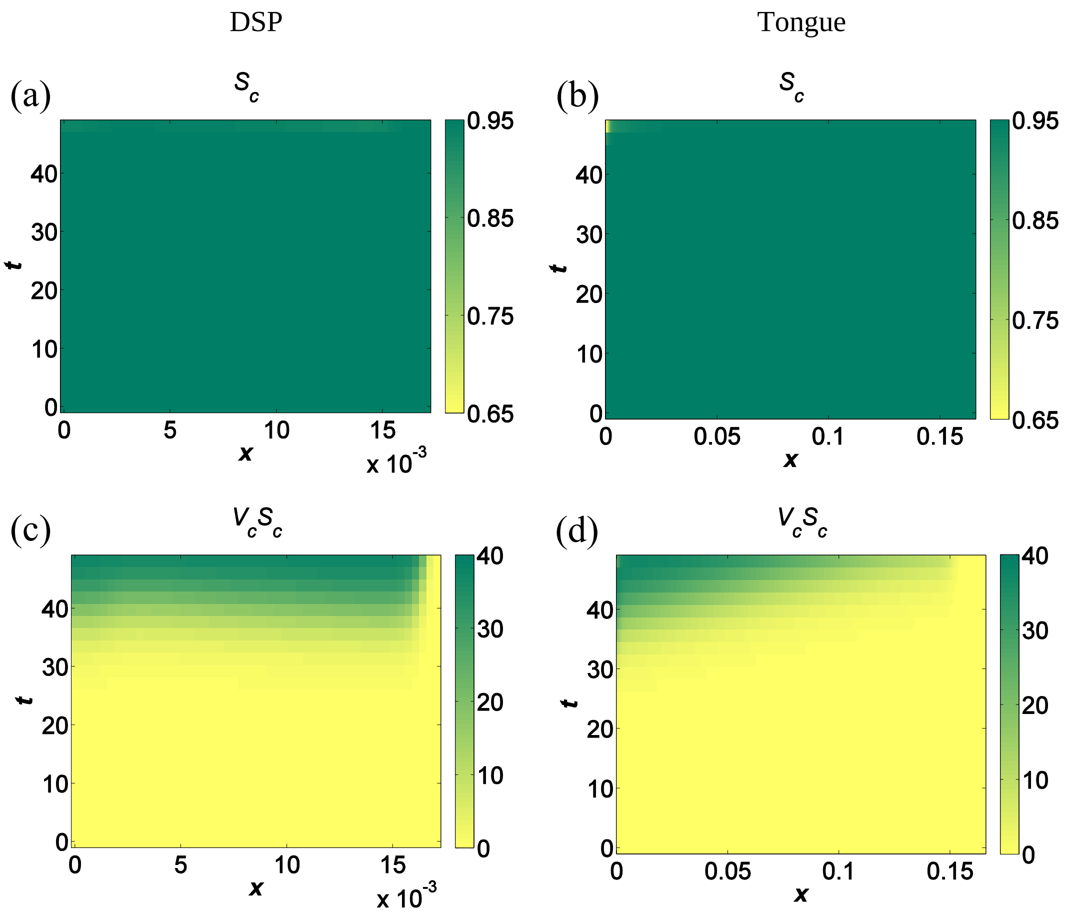

Fig. S5.2. Simulation results for altered viral replication and uptake parameter estimates. Results for DSP and tongue over a 48 hour timescale for maximal replication rate, ξ, and rate of FMDV resource consumption, ρ, both set at $0.29\times$ their default estimates. Viral uptake by the spinous layer is defined here as half that for the basal layer. Epithelium surface used as the viral entry point for DSP and basement membrane as the viral entry point for tongue. (a), (b) Cellular space fraction, S_c, of DSP and tongue respectively. Reduced levels of cellular space fraction are observed at the top left hand corner of graph (b). (c), (d) Intracellular virus load, V_cS_c, of DSP and tongue respectively measured in PFU/cm.

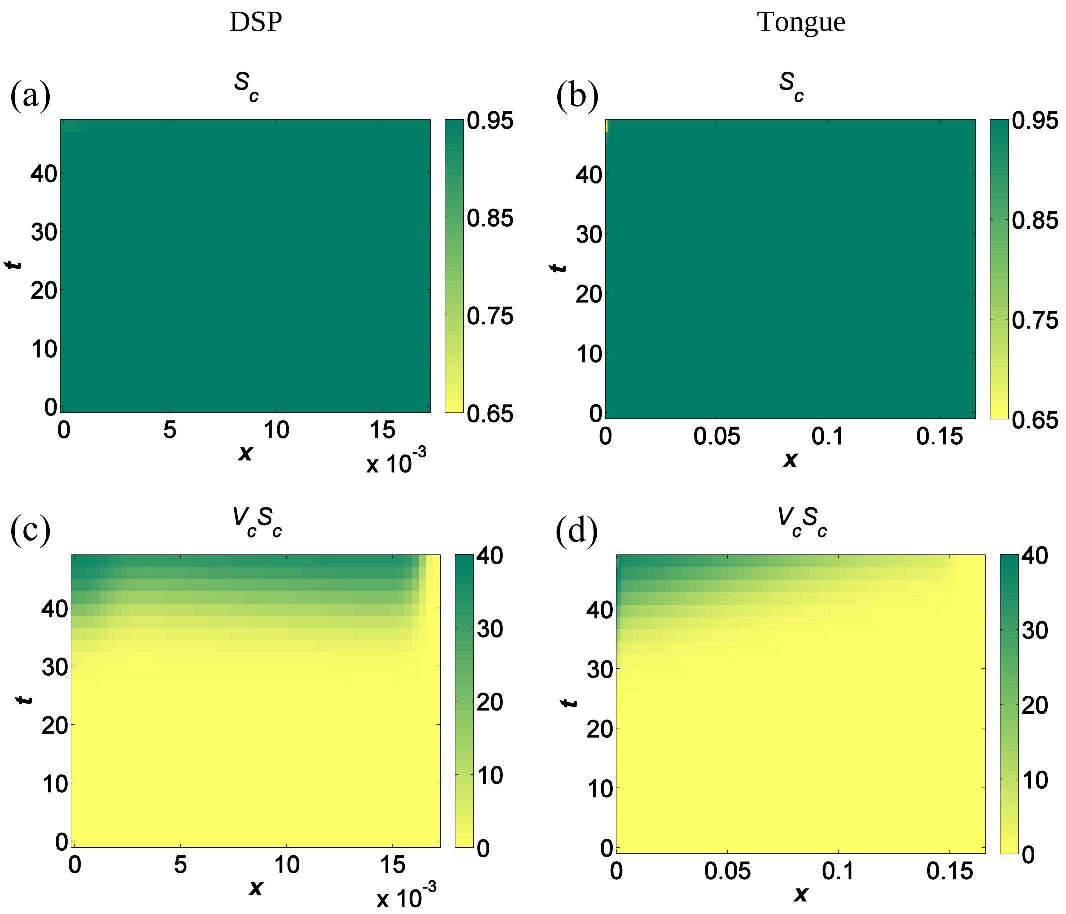

Fig. S5.3. Simulation results for altered viral replication and uptake parameter estimates. Results for DSP and tongue over a 48 hour timescale for maximal replication rate, ξ, and rate of FMDV resource consumption, ρ, both set at $0.29\times$ their default estimates. Viral replication in the spinous layer and viral uptake by the same layer are defined here as half of that for the basal layer. Epithelium surface used as the viral entry point for DSP and basement membrane as the viral entry point for tongue. (a), (b) Cellular space fraction, S_c, of DSP and tongue respectively. Reduced levels of cellular space fraction are observed at the top left hand corner of graph (b). (c), (d) Intracellular virus load, V_cS_c, of DSP and tongue respectively measured in PFU/cm.

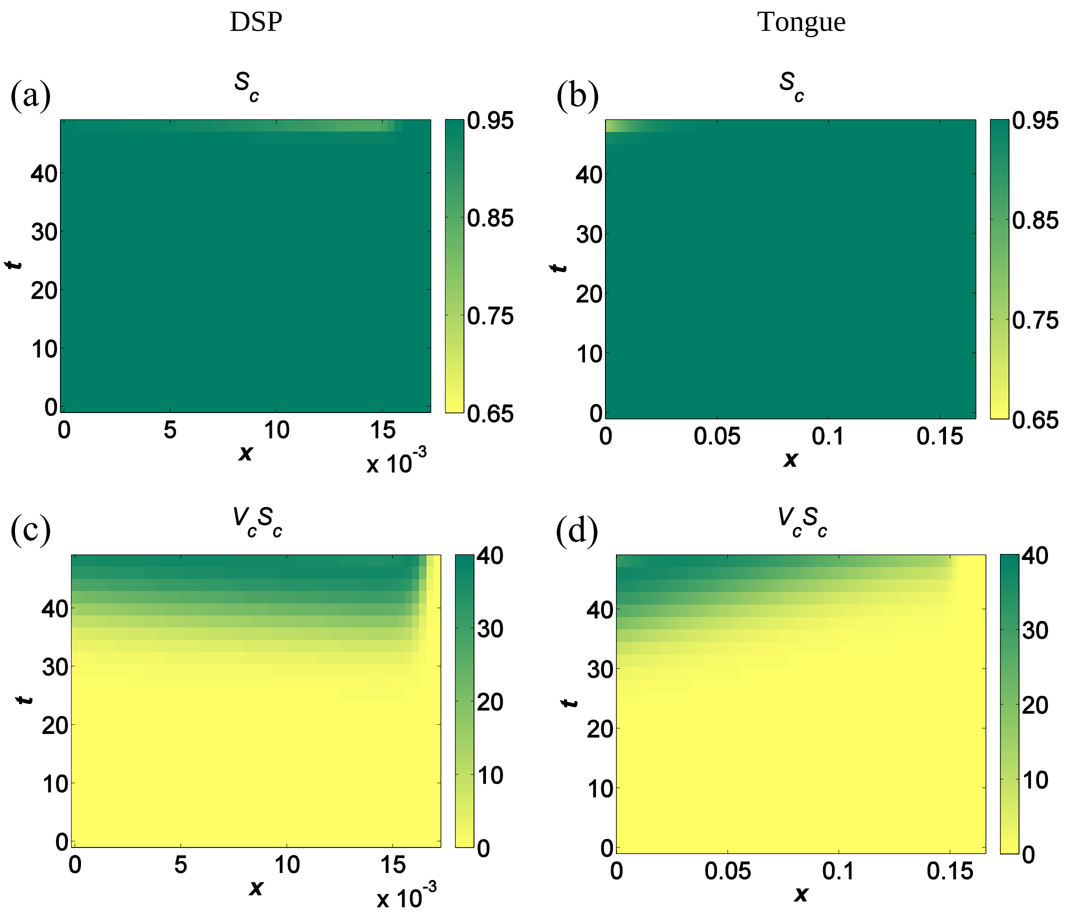

Fig. S5.4. Simulation results for altered viral replication and mass transfer parameter estimates. Results for DSP and tongue over a 48 hour timescale for maximal replication rate, ξ, and rate of FMDV resource consumption, ρ, both set at $0.29\times$ their default estimates. FMDV mass transfer coefficient (non-dimensional), Q_V, is increased to 7. Epithelium surface used as the viral entry point for DSP and basement membrane as the viral entry point for tongue. (a), (b) Cellular space fraction, S_c, of DSP and tongue respectively. (c), (d) Intracellular virus load, V_cS_c, of DSP and tongue respectively measured in PFU/cm.

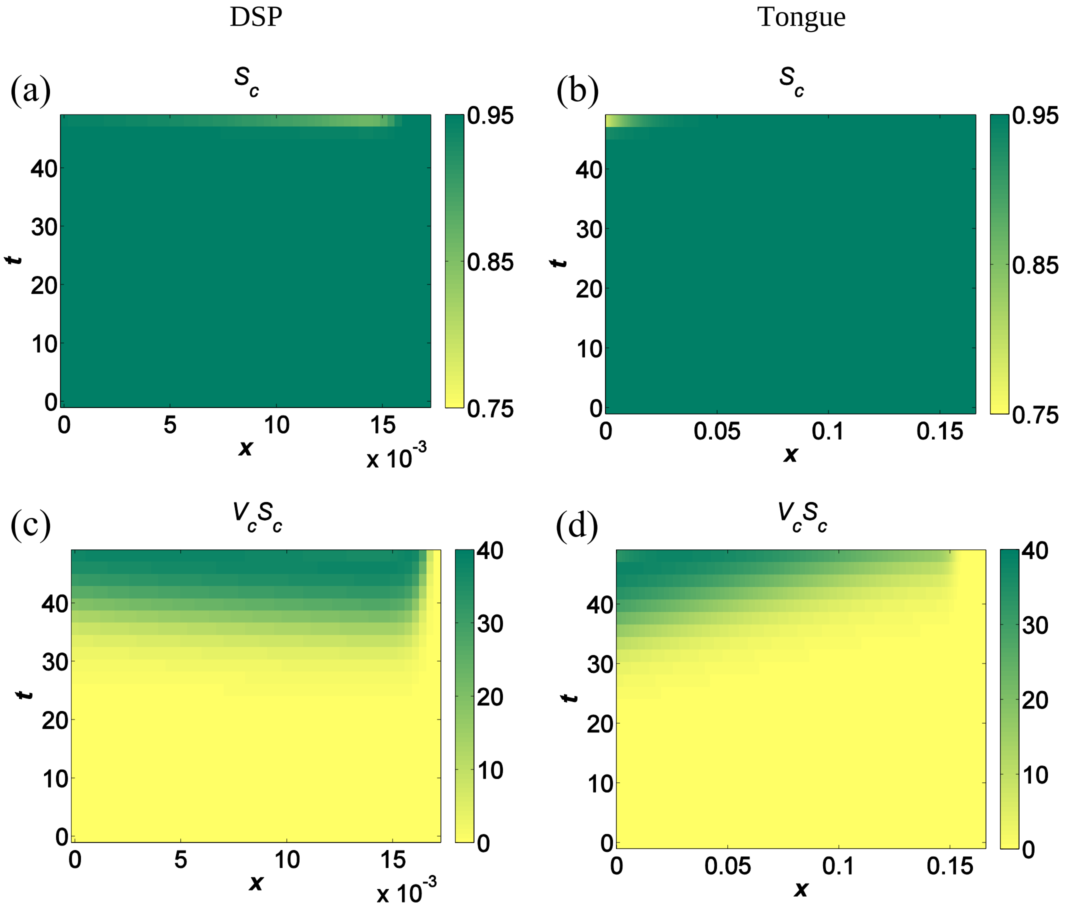

Fig. S5.5. Simulation results for altered viral replication and intracellular resource parameter estimates. Results for DSP and tongue over a 48 hour timescale for maximal replication rate, ξ, and rate of FMDV resource consumption, ρ, both set at $0.29\times$ their default estimates. The threshold concentration of resource (non-dimensional), $K_{1/2}$, is set to 0.03. Epithelium surface used as the viral entry point for DSP and basement membrane as the viral entry point for tongue. (a), (b) Cellular space fraction, S_c, of DSP and tongue respectively. (c), (d) Intracellular virus load, V_cS_c, of DSP and tongue respectively measured in PFU/cm.
